# Hydrogen Sulfide in Balneology: Physiology, Evidence, and Clinical Translation

**DOI:** 10.3390/ijms262110790

**Published:** 2025-11-06

**Authors:** Jose Manuel Carbajo, Francisco Maraver, Lorena Vela, Constantin Munteanu

**Affiliations:** 1 Medical Hydrology Group, Department of Radiology and Rehabilitation, Complutense University of Madrid, Plaza Ramón y Cajal s/n, 28040 Madrid, Spain; jocarbaj@ucm.es (J.M.C.); marialorenavela@ucm.es (L.V.); 2 Professional School of Medical Hydrology, Complutense University of Madrid, Plaza Ramón y Cajal s/n, 28040 Madrid, Spain; 3 Faculty of Medical Bioengineering, University of Medicine and Pharmacy “Grigore T. Popa” Iași, 700115 Iași, Romania; constantin.munteanu.biolog@umfiasi.ro

**Keywords:** hydrogen sulfide, sulfurous waters, balneotherapy, dermatology, rheumatology, inhalation therapy, spa therapy, sulfur springs, peloids, epigenetics

## Abstract

This review integrates the biology and clinical translation of hydrogen sulfide (H_2_S) in balneology. It frames H_2_S as a gasotransmitters with dual chemical and biological actions and summarizes the H_2_S/HS^−^ equilibrium as a function of pH, temperature, and oxygenation, which governs bioaccessibility in sulfurous waters. Endogenous and exogenous sources, transport, and mitochondrial catabolism are outlined, together with core cellular mechanisms: protein persulfidation; activation of Nrf2/ARE; modulation of NF-κB; regulation of ion channels; and engagement of PI3K/Akt, MAPK/ERK, and Wnt pathways, plus epigenetic interactions with HDACs and sirtuins. Preclinical and clinical evidence in dermatology, musculoskeletal disease, and respiratory care is synthesized, alongside metabolic, cardiovascular, gastrointestinal, and renal effects. Technical aspects that preserve the bioactive fraction of H_2_S while meeting environmental safety limits are highlighted. Routes of administration (bathing, peloids, inhalation, and drinking cures) and key operational parameters are described. Overall, the review links physicochemical and molecular foundations with clinical indications for sulfurous waters and derivatives and identifies opportunities for research and development in H_2_S donors and thermal cosmetics without extrapolating beyond the available data.

## 1. Introduction

Hydrogen sulfide (H_2_S), previously considered merely a toxic byproduct of anaerobic metabolism, is now recognized as an endogenous gasotransmitter with important physiological functions, comparable to nitric oxide (NO) and carbon monoxide (CO) [1,2].

Over the past two decades, H_2_S has been identified as a critical modulator of cellular signaling, redox homeostasis, inflammation, and epigenetic modulation with an increasing interest in its therapeutic applications, particularly in the context of balneotherapy with sulfurous medicinal waters [1,3,4]. Recent years have witnessed a surge in discoveries related to the signaling roles of polysulfides and their superior redox potential compared to H_2_S, prompting reconsideration of therapeutic sulfur species in balneology. In parallel, novel H_2_S delivery technologies and deeper insights into mitochondrial targets such sirtuins, have advanced our mechanistic understanding and opened translational avenues for sulfur-rich interventions in clinical and wellness contexts [5].

Accumulating evidence indicates that H_2_S contributes to cutaneous, musculoskeletal, and vascular health through mechanisms involving ion channel modulation, persulfidation of cysteine residues, and regulation of transcriptional networks such as the Nrf2, NF-κB, and sirtuin pathways. In this context, sulfur-rich mineral waters have shown significant therapeutic promise in dermatological and rheumatologic disorders, with mechanistic underpinnings increasingly supported by molecular and translational research [3,6].

The present review provides an updated, integrative synthesis of the biochemistry, molecular targets, cellular effects, and clinical applications of H_2_S, with a specific focus on its role in balneological medicine. In doing so, it aims to bridge basic redox biology with practical therapeutic implementations, contextualized by emerging technologies for delivery, monitoring, and clinical validation of H_2_S-based interventions.

## 2. Nature of Hydrogen Sulfide (H_2_S)

H_2_S is a colorless gas with a characteristic “rotten egg” odor. Its pKa1 is 7.0, and at physiological pH, it coexists as H_2_S and HS^−^ [7]. It can cross biological membranes, facilitating its intracellular action [8]. In aqueous environments, its equilibrium depends on pH, temperature, the concentration of other ions, and the presence of dissolved oxygen [9].

### 2.1. Physicochemical Properties

From a balneological standpoint, the concentration of H_2_S in water depends on three critical factors: pH, temperature, and oxygenation. At neutral or slightly acidic pH and low oxygenation, the equilibrium favors the presence of dissolved H_2_S, which is the most bioavailable and lipophilic form [3,4]. Higher temperatures favor gas volatilization, increasing its availability for inhalation treatments, but reducing its persistence in topical applications, through the skin or ingestion, if not properly controlled [10].

From a physicochemical perspective, hydrogen sulfide (H_2_S) is a gas with high solubility in water, which allows it to disperse easily in aqueous media. Additionally, it behaves as a weak diprotic acid and, when dissolved, establishes a dynamic equilibrium between different chemical species [7].

In aqueous solution, H_2_S coexists with its dissociated forms: the hydrosulfide anion (HS^−^) and the sulfide anion (S^2−^). The undissociated molecular form, H_2_S, is lipophilic, which allows it to pass through biological membranes by passive diffusion, and it is considered the most biologically active fraction [7].

This equilibrium is determined by its dissociation constants:

- pKa_1_ ≈ 6.9 → H_2_S ⇌ H^+^ + HS^−^.
- pKa_2_ ≈ 12 → HS^−^ ⇌ H^+^ + S^2−^.

The gaseous form of H_2_S, essential for cutaneous and respiratory absorption, is particularly sensitive to hydrothermal variables, as described below, Table 1:

- **Water pH**: A pH between 5.5 and 6.5 favors the presence of molecular H_2_S, facilitating its absorption by passive diffusion. As pH becomes more alkaline, bioavailability decreases due to conversion into HS^−^, which is less bioavailable via transcutaneous or respiratory routes [10].
- **Temperature**: Increased temperature decreases the solubility of H_2_S in water, promoting its transition to the gaseous phase. This enhances its inhalation bioavailability but also accelerates volatilization, reducing its effective concentration in baths [3].
- **Dissolved oxygen**: H_2_S is rapidly oxidized to thiosulfate, sulfite, or sulfate in the presence of oxygen, reducing its biological activity, especially at elevated temperatures. Thus, hypoxic environments favor its preservation in active form, as demonstrated by water analyses and direct capture techniques in thermal environments. Therefore, the lower the dissolved oxygen content—avoiding bubbles and microbubbles—the more stable the H_2_S remains in its reduced and therapeutic form. In spas, water retention in pools, recirculation, or atmospheric exposure also significantly influences H_2_S loss. Hence, thermal circuit design should minimize aeration and turbulence to achieve the highest concentration of gaseous hydrogen sulfide [10].

These considerations underscore the need for careful technical management in spa facilities, where preservation of active H_2_S depends on:

- The pH of sulfurous mineral water.
- Controlled temperatures.
- Minimization of aeration and excessive recirculation.
- Use of techniques that limit volatilization losses.

Understanding this dynamic allows for the design of appropriate protocols to preserve or eliminate the therapeutic gaseous fraction of H_2_S needed to ensure its safe application in dermatological, rheumatological, respiratory, and digestive disorders [10].

From an engineering standpoint, therapeutic pools that aim to preserve dissolved H_2_S typically employ: (i) short water-retention times (≤30 min turnover) to minimize oxygen ingress, (ii) bottom-fed laminar flow inlets that displace water upward with minimal turbulence, (iii) overflow gutters or weirs designed to skim only the uppermost centimeter where volatilization is greatest, and (iv) closed-loop recirculation with deaerated head-spaces or floating thermal blankets. Successful examples include the low-velocity ‘silent flow’ systems at Terme di Sirmione (Italy) and the semi-covered, hypoxic basins at Techirghiol Balneary Resort (Romania), both of which maintain free H_2_S concentrations >0.9 mg L^−1^ at 34 °C while keeping workplace air levels below 5 ppm [10,11,12]. Integration of real-time redox sensors and variable-speed pumps has further allowed operators to adjust flow architecture dynamically in response to changes in pH, temperature, and bather load, ensuring consistent therapeutic dosing without exceeding safety thresholds.

### 2.2. Endogenous and Exogenous Sources

The biosynthesis of hydrogen sulfide (H_2_S) in human tissues has been well described by Olson [7] and reviewed by Kimura [13]. The body produces H_2_S in its anabolic metabolism through three main enzymes: cystathionine β-synthase (CBS), cystathionine γ-lyase (CSE), and 3-mercaptopyruvate sulfurtransferase (3-MST). Cysteine aminotransferase (CAT) also plays a role [14,15], acting on L-cysteine in tissues such as the liver, brain, endothelium, and skin [16], Table 2.

Exogenously, H_2_S with therapeutic purposes is found in sulfurous medicinal waters and their derivatives, in the diet, and in slow-releasing sulfur compounds [18,19].

Sulfurous mineral waters are a natural and topical source of H_2_S, fundamental in balneotherapy, especially in dermatology and rheumatology [11,12]. As highlighted, the most active form is dissolved hydrogen sulfide (H_2_S), in equilibrium with sulfurous ions (HS^−^ and S^2−^), depending on pH.

The bacterial fermentation of sulfur-rich proteins (cysteine, methionine) by the intestinal microbiota generates H_2_S as a metabolite [20]. Sulfate-reducing bacteria (e.g., *Desulfovibrio*, *Bilophila wadsworthia*) are responsible for this synthesis, and colonic H_2_S can modulate the intestinal epithelium and exert pro- or anti-inflammatory effects depending on dose, potentially affecting several organs [21].

The biological impact of colonic H_2_S is concentration- and compartment-dependent. In the luminal phase (~0.3–2 mM), H_2_S functions as an electron sink that supports anaerobic energy metabolism and, at low micromolar diffusion into epithelial cells, activates Nrf2-mediated antioxidative responses and tight-junction reinforcement—actions generally regarded as protective [20]. Conversely, high-protein diets or dysbiosis can push mucosal H_2_S exposure above 500 µM, overwhelming mitochondrial SQR detoxification in colonocytes, inhibiting butyrate oxidation, and triggering DNA damage pathways, thereby shifting toward a pro-inflammatory/cytotoxic profile [22].

Importantly, the interplay between H_2_S and short-chain fatty acids (SCFAs) modifies this dichotomy. Butyrate up-regulates colonic SQR and enhances epithelial oxygen consumption, thereby increasing the detoxification threshold for H_2_S, whereas sulfide-overload suppresses butyrate β-oxidation, creating a feed-forward loop that favors sulfate-reducing bacteria (SRB) expansion. Dietary fiber patterns that elevate luminal butyrate—e.g., inulin-type fructus or resistant starch—thus tilt the balance toward a tolerogenic milieu, providing a mechanistic rationale for combining SAA-controlled diets with prebiotic supplementation in spa rehabilitation programmers [23,24].

Currently, experimental drugs and nutraceuticals that release H_2_S in a controlled manner have been synthesized, such as NaHS, GYY4137, diallyl trisulfide (garlic), and sulforaphane (broccoli), with researched applications in hypertension, neurodegeneration, chronic inflammation, and cancer [9,25].

Dietary supply of sulfur amino acids (SAAs) is the principal systemic precursor pool for endogenous H_2_S synthesis. Current FAO/WHO “Food and Agriculture Organization/World Health Organization” recommendations set a combined cysteine + methionine requirement of ≈13 mg kg^−1^ day^−1^ for healthy adults, yet typical Western diets deliver 25–35 mg kg^−1^ day^−1^—roughly triple the basal need [26].

### 2.3. Transport, Catabolic Metabolism, and Excretion of Hydrogen Sulfide

Hydrogen sulfide (H_2_S) gas is a highly lipophilic molecule, which allows it to passively diffuse through the skin and cell membranes. However, depending on its chemical form, it circulates more or less effectively.

At physiological pH (~7.4), the equilibrium shifts toward HS^−^ (80%) and H_2_S (20%), while S^2−^ is practically nonexistent. The gaseous fraction of H_2_S is mainly bound in blood to plasma proteins such as hemoglobin, which transports it and regulates its availability. The ionic forms (HS^−^) have low cutaneous and pulmonary absorption capacity and are transported dissolved in blood plasma. Therefore, exogenous hydrosulfides absorbed into the bloodstream primarily originate from the transformation of absorbed gaseous sulfides, which are converted into hydrosulfides due to physiological blood pH [27].

Cellular absorption of hydrogen sulfide (H_2_S) varies significantly depending on physiological conditions, especially pH and temperature [10].

In the lungs, where extracellular pH is approximately 7.2–7.4 and body temperature is about 37 °C, the acid-base equilibrium shifts H_2_S toward its dissociated form: the hydrosulfide anion (HS^−^) accompanied by a proton (H^+^). This ionic form cannot freely cross the lipid bilayer of cell membranes, so its transport depends on specific mechanisms such as the AE1 (Anion Exchanger 1), which facilitates the entry of HS^−^ into the cell by exchanging it with other anions like chloride (Cl^−^). AE1 (SLC4A1) is expressed mainly in erythrocytes and renal intercalated cells and therefore contributes little to epithelial uptake of HS^−^. In airway, intestinal, and skin epithelia, available evidence implicates members of the SLC26 family—particularly SLC26A3 (DRA), SLC26A6 (PAT1), and pendrin (SLC26A4)—in HS^−^/Cl^−^ or HS^−^/HCO_3_^−^ exchange, providing an alternative route for epithelial sulfide transport [28]. This transport is slower, regulated by electrochemical gradients, and susceptible to saturation or inhibition, limiting the efficiency of H_2_S absorption in these tissues.

In contrast, the skin has a notably different environment. Its extracellular pH, particularly in the stratum corneum, ranges from 4.5 to 6.0, with a slightly lower temperature of 34–35 °C. Under these conditions, H_2_S is predominantly in its neutral, gaseous form. This is key because it allows H_2_S to diffuse directly through cell membranes via passive diffusion, without the need for transporters or energy expenditure. This diffusion, driven by the concentration gradient, enables rapid and efficient entry of the gas between skin cell layers. Kimura [13], along with other authors such as Kabil and Banerjee [8] and Olson [7], showed that the ability of H_2_S to act as a gasotransmitters is directly related to its chemical form and the acidic nature of the surrounding tissue.

**Figure 1:**
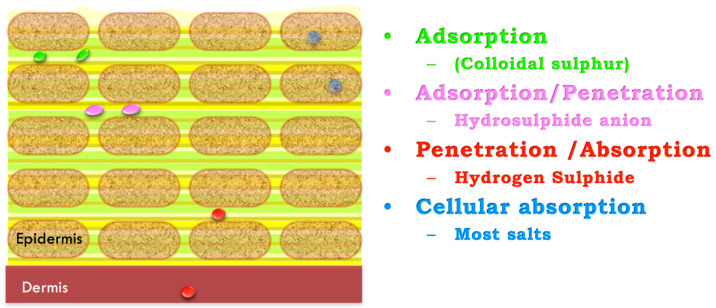
Adsorption/penetration/absorption capacity of water-soluble or water-dispersible solutes, hydrogen sulfide behavior [29].

Thus, skin physiology not only permits but optimizes cellular absorption of hydrogen sulfide, reinforcing its role as a key therapeutic target in treatments involving sulfurous waters or topical H_2_S donors Figure 1.

Hydrogen sulfide (H_2_S) is primarily eliminated through mitochondrial oxidation, a highly regulated process that occurs in tissues with high mitochondrial density, such as the liver, kidney, brain, and cardiac muscle [3].

The main degradation pathway of H_2_S begins with the action of the enzyme sulfide quinone oxidoreductase (SQR), located in the inner mitochondrial membrane. This enzyme catalyzes the initial oxidation of H_2_S, transferring a sulfur atom to a cysteine residue of an acceptor protein to form a persulfide. This reaction not only initiates H_2_S detoxification but also channels its electrons toward ubiquinone (coenzyme Q10), integrating it partially into the mitochondrial respiratory chain [30].

Next, the generated persulfide groups are oxidized by the enzyme ETHE1 (ethylmalonic encephalopathy protein), a soluble mitochondrial dioxygenase that converts these compounds into sulfites (SO_3_^2−^). The sulfite may follow two routes: conversion to thiosulfate (S_2_O_3_^2−^) or transformation into sulfate (SO_4_^2−^) by sulfite oxidase. The sulfate, fully oxidized and water-soluble, represents the final form of H_2_S elimination, being excreted renally [31].

Beyond its detoxifying role, this oxidative process contributes significantly to ATP production, especially at low H_2_S concentrations. In this context, H_2_S acts as an alternative energy substrate, capable of partially fueling the electron transport chain, which has led to its consideration as an energy source of physiological and pathological relevance [32].

After its endogenous or exogenous production, hydrogen sulfide (H_2_S) is rapidly catabolized and eliminated by the body to prevent toxic accumulation. The predominant elimination route is oxidation in the liver and, to a lesser extent, in the kidney, generating inorganic sulfate (SO_4_^2−^), which is mostly excreted in urine. This route constitutes the main final destination of H_2_S and represents an efficient detoxification and homeostatic control mechanism for tissue levels [1,32].

A second relevant elimination pathway, especially in clinical and toxicological contexts, is the partial conversion of H_2_S into thiosulfate (S_2_O_3_^2−^), which is also excreted in urine. Thiosulfate, an intermediate product of H_2_S oxidation, is commonly used as a biological biomarker for recent gas exposure, particularly in occupational settings or cases of acute poisoning [33].

Additionally, a small fraction of H_2_S can be eliminated unmetabolized. This lipophilic gas can diffuse through biological membranes and be exhaled through the lungs. In smaller amounts, it can also be excreted via sweat or feces, especially in cases of overload or external exposure. The efficiency of these catabolic pathways influences the toxicokinetic of H_2_S, its potential accumulation in mitochondrial or hepatic dysfunction, and its local or systemic effects depending on tissue concentration.

## 3. Physiological Mechanisms of H_2_S: Chemical and Biological Activity

H_2_S is an endogenous gas with both chemical (direct antioxidant or cysteine persulfidation) and biological activity (modulation of enzymes, ion channels, and mitochondria). It acts as a short-term antioxidant by neutralizing free radicals and activating enzymes such as superoxide dismutase. In the long term, it exerts epigenetic effects by inhibiting histone deacetylases (HDACs) and promoting the expression of antioxidant genes. Physiologically, it participates in vasodilation, neuroprotection, immune regulation, and cellular homeostasis.

### 3.1. Chemical Mechanism: Antioxidant Activity [Scavengers]

Hydrogen sulfide (H_2_S) is recognized as a gasotransmitter with potent antioxidant capacity, both through direct action on reactive species and by modulating cellular redox pathways. This antioxidant property is not only due to the H_2_S molecule itself but also to its oxidized intermediates, particularly polysulfides (H_2_Sₙ, n ≥ 2), whose chemistry and biological activity are gaining increasing attention. Polysulfides are generally generated by the reaction of hydrogen sulfide with hydrogen peroxide.

Initially, H_2_S acts as a direct antioxidant by neutralizing reactive oxygen species (ROS), such as hydrogen peroxide (H_2_O_2_), superoxide anion (O_2_•^−^), and hydroxyl radical (•OH), thereby reducing oxidative cellular damage [2,13]. Simultaneously, it exerts an indirect effect by modulating endogenous antioxidant systems, inducing the expression and activity of enzymes such as superoxide dismutase (SOD), glutathione peroxidase (GPx), catalase, and increasing intracellular levels of reduced glutathione (GSH) [4].

In biological environments, H_2_S can oxidize in the presence of oxygen or reactive species (free radicals) to form polysulfides, molecules that contain linear sulfur atom chains (e.g., H_2_S_2_, H_2_S_3_, H_2_S_4_). These compounds exhibit greater redox capacity than H_2_S itself and can act as more potent donors of reduced sulfur (S^0^) and persulfurated species [34].

Polysulfides display increased reactivity toward nucleophiles with free thiol groups (–SH), more efficiently generating protein persulfidation than H_2_S alone. This protective post-translational modification, also called S-sulfuration, preserves the thiol groups of cysteine residues from irreversible oxidation and regulates the function of numerous proteins involved in redox homeostasis and cellular signaling [35].

Notably, polysulfides also activate Nrf2 more effectively than H_2_S, enhancing the expression of cytoprotective genes, particularly through the persulfidation of cysteine residues in key proteins, modulating their function.

The primary mechanism of H_2_S action is persulfidation or the addition of an –SSH group to cysteine residues of target proteins. This post-translational change alters the structure, activity, or subcellular localization of the protein [35,36], greatly influencing the Nrf2/Keap1 pathway (nuclear factor erythroid 2-related factor 2), which increases gene expression of antioxidant enzymes as further detailed. Nrf2 activation, once translocated to the nucleus in response to H_2_S, promotes transcription of antioxidant genes such as heme oxygenase-1 (HO-1) and NQO1 [37].

Persulfidation (S-sulfuration) also significantly influences ion channels, especially relevant in the cardiovascular and nervous systems. This interaction is crucial for processes such as vasodilation, neurotransmission, oxidative stress response, and the regulation of vascular tone and blood pressure.

H_2_S directly activates K_ATP channels present in the membrane of vascular smooth muscle cells, inducing membrane hyperpolarization and thus smooth muscle relaxation. This effect leads to vasodilation, reduced peripheral resistance, and lower blood pressure. The sulfurization (persulfidation) of cysteine residues in potassium channel subunits, such as Kir6.1 and SUR2B, also alters their activity [38]. These channels are involved in the regulation of vascular tone, insulin secretion, and other cellular functions.

Not only are K^+^ channels influenced—so are large-conductance calcium-activated potassium channels (BK_Ca), also known as “Big Potassium” or KCa1.1. Their role includes inhibition of calcium entry via L-type channels, sodium and chloride channels, and TRP (Transient Receptor Potential) channels, especially TRPV1 and TRPA1, which are important in nociception, pain perception, and neuroinflammatory responses [39].

This illustrates how hydrogen sulfide acts via two mechanisms: an immediate chemical–molecular action, and a longer-lasting effect by interfering with gene expression processes—i.e., by influencing cellular epigenetics. The activity of polysulfides is not merely chemical, but also functional. In the skin, it has been proposed that the therapeutic effect of many H_2_S-rich sulfurous waters may partly be due to the formation of polysulfides in the stratum corneum and skin surface, where they exert a prolonged local antioxidant effect. Additionally, polysulfides may cross cell membranes more easily, acting as active sulfur transport forms and prolonging intracellular antioxidant signaling [40].

Both H_2_S and polysulfides modulate mitochondrial bioenergetics by improving the efficiency of the respiratory chain and limiting the production of mitochondrial ROS [8]. They also demonstrate functional synergy with nitric oxide (NO), forming bioactive nitrososulfur species such as HSNO and SSNO^−^, which expand the range of antioxidants and vasodilatory signaling [36].

Consequently, their role as functional intermediaries of H_2_S is essential to understanding the true scope of its antioxidant effects [40]. The antioxidant capacity of H_2_S is therefore not limited to its role as a direct reducing molecule but is amplified and diversified through its conversion into biologically active polysulfides. These species not only possess greater reactivity and efficacy against oxidative damage but also act as potent signaling agents, redox regulators, protein modulators, and defenders of cellular integrity. Altogether, the H_2_S–polysulfide system represents a versatile endogenous defense and a promising strategy for redox-based therapies Table 3.

### 3.2. Biological Mechanisms: Cellular Signaling

The biological activity of hydrogen sulfide (H_2_S) at the cellular level is exerted at low concentrations through direct molecular mechanisms—such as post-translational modification of proteins via persulfidation (S-sulfuration)—and indirect mechanisms, such as the modulation of cell signaling pathways, redox control, or epigenetic regulation.

In addition to the direct antioxidant action described earlier, and the indirect induction of antioxidant enzymes, H_2_S also modulates intracellular targets including hemoproteins (e.g., cytochrome c oxidase), iron-sulfur and zinc-sulfur protein clusters, and especially various ion channels—mainly ATP-sensitive potassium (K_ATP) channels. Furthermore, it influences genetic pathways involved in inflammation, antioxidant responses, and cell survival.

In the long term, its epigenetic role is carried out through inhibition of histone deacetylases (HDACs) and regulation of microRNAs, impacting gene expression related to inflammation, cell proliferation, and senescence.

Thus, H_2_S functions as a multifaceted physiological modulator, integrating metabolic, redox, and epigenetic signals across multiple tissues, with particular relevance in the cardiovascular, nervous, and immune systems.

#### 3.2.1. Nrf2/Keap1 Pathway

The interaction between hydrogen sulfide (H_2_S) and the Nrf2/Keap1 pathway represents one of the key mechanisms by which this gasotransmitter exerts cytoprotective, antioxidant, and anti-inflammatory effects [41].

H_2_S modulates the cellular antioxidant response primarily by activating the Nrf2 (nuclear factor erythroid 2–related factor 2) pathway. Under normal conditions, Nrf2 is sequestered in the cytoplasm by Keap1 (Kelch-like ECH-associated protein 1), which promotes its degradation via the proteasome. However, H_2_S can modify this interaction through a persulfidation (S-sulfuration) process of cysteine residues on Keap1 [37].

This modification induces a conformational change in Keap1 that prevents Nrf2 ubiquitination, allowing it to accumulate and translocate into the nucleus. Once in the nucleus, Nrf2 binds to antioxidant response elements (AREs) and induces the transcription of cytoprotective genes such as: HO-1 (heme oxygenase-1); NQO1 (NAD(P)H: quinone oxidoreductase 1); GPx (glutathione peroxidase); SOD (superoxide dismutase) and γ-GCS (glutamate–cysteine ligase) [42].

This H_2_S–Keap1/Nrf2–antioxidant gene axis constitutes a key defense mechanism against oxidative stress, inflammation, and ROS-induced apoptosis.

Additionally, H_2_S may enhance Nrf2 activity indirectly by reducing mitochondrial ROS levels and regulating upstream pathways such as PI3K/Akt, which also stabilize Nrf2.

#### 3.2.2. PI3K/Akt/mTOR Pathway

Among the major cell signaling pathways, the PI3K/Akt/mTOR (phosphatidylinositol 3-kinase/protein kinase B/mammalian target of rapamycin) axis plays a central role in regulating cellular proliferation, survival, metabolism, and repair.

Hydrogen sulfide (H_2_S) can modulate the PI3K/Akt/mTOR pathway through redox-dependent and post-translational mechanisms, particularly by activating PI3K/Akt. H_2_S stimulates Akt phosphorylation at Ser473 and Thr308, promoting its activation. This effect has been observed in endothelial, neuronal, hepatic, and tumor cells, with physiological or pathological outcomes depending on context:

- Under oxidative stress, H_2_S promotes cell survival by activating PI3K/Akt and reducing ROS via the regulation of antioxidant enzymes such as SOD, catalase, or GPx [37].
- In the cardiovascular system, H_2_S stimulates the PI3K/Akt pathway to protect against ischemia–reperfusion injury, decreasing apoptosis and mitochondrial damage [43].

Akt activation indirectly leads to the activation of mTORC1, which regulates protein synthesis, autophagy, and cell metabolism:

- In mesenchymal stem cells, H_2_S promotes proliferation and osteogenic differentiation by activating mTOR, thereby enhancing regenerative processes [44].
- In certain tumor models, however, mTOR inhibition by H_2_S may produce antiproliferative and pro-autophagic effects, suggesting a biphasic action dependent on dose and cell type [45].

H_2_S exerts post-translational modifications such as S-sulfuration of cysteine residues in regulatory proteins of this pathway, directly modulating the enzymatic activity of PI3K, PTEN, or mTOR, thus influencing the balance between cell growth and apoptosis [4].

This pathway presents promising therapeutic and translational applications in neuroprotection, regenerative dermatology and wound healing, and inflammatory diseases such as ulcerative colitis or arthritis.

#### 3.2.3. Wnt/β-Catenin Pathway

The Wnt/β-catenin signaling pathway is essential for regulating cell proliferation, differentiation, regeneration, and tissue homeostasis, particularly in the skin, intestinal tract, and nervous system. Hydrogen sulfide (H_2_S) can modulate this pathway in various ways, with effects that depend on cellular context, redox environment, type of stimulus, and H_2_S concentration. There is no uniform effect of H_2_S on Wnt/β-catenin activity.

Wnt proteins are a family of secreted extracellular signaling glycoproteins that activate membrane receptors. This activation inhibits the degradation complex of the intracellular protein β-catenin (composed of Axin, APC, GSK-3β, and CK1), allowing β-catenin to accumulate and translocate to the nucleus. In the nucleus, β-catenin binds to TCF/LEF transcription factors (T-cell factor/Lymphoid enhancer-binding factor), regulating the transcription of genes involved in cell proliferation, migration, and differentiation [46].

H_2_S may activate or modulate the Wnt/β-catenin pathway through several mechanisms: Via persulfidation (S-sulfuration) of pathway regulatory proteins, modifying their activity and stability; By modulating GSK-3β, an inhibitory kinase of β-catenin, promoting its stabilization and nuclear translocation and By enhancing tissue regeneration, where H_2_S has been shown to potentiate the MAPK/ERK pathway [47], contributing to cross-talk between Wnt signaling and other proliferative cascades.

Recent in vitro and in vivo studies in hepatocellular carcinoma models have demonstrated that the slow-releasing H_2_S donor GYY4137 significantly inhibits the phosphorylation of GSK-3β and β-catenin, thereby downregulating the AKT/GSK-3β/β-catenin pathway and promoting apoptosis in tumor cells [48]. Systemic administration of the slow-releasing H_2_S donor GYY4137 significantly enhanced ferroptosis-based tumor suppression in non-small cell lung cancer (NSCLC) models, particularly under cystine-depleted conditions. Mechanistically, GYY4137 promoted the persulfidation of S-adenosylhomocysteine hydrolase (SAHH) at Cys195, inhibiting its enzymatic activity, reducing homocysteine levels, and consequently depleting intracellular cysteine and glutathione. This metabolic shift sensitized NSCLC cells to ferroptosis both in vitro and in vivo, reinforcing the broader role of H_2_S donors as metabolic modulators with therapeutic potential beyond superficial tissue contexts [49].

#### 3.2.4. MAPK/ERK Pathway

The MAPK/ERK (Mitogen-Activated Protein Kinase/Extracellular Signal-Regulated Kinase) pathway is one of the most important intracellular cascades involved in cell proliferation, differentiation, survival, and stress response. Hydrogen sulfide (H_2_S) can modulate this pathway in a complex and context-dependent manner, with both activating and inhibitory effects depending on the cell type, concentration, and exposure time.

H_2_S can transiently activate the ERK1/2 pathway through redox-related mechanisms, such as S-sulfuration of regulatory proteins, enhancing signaling. Conversely, it may inhibit sustained ERK activation under oxidative stress conditions, thereby reducing cellular damage. Mechanistically, persulfidation of Ras at Cys118 and inhibition of the upstream phosphatase MKP-1 have been identified as redox checkpoints for ERK activation. In a murine model of diabetic nephropathy, systemic administration of the slow-releasing H_2_S donor GYY4137 attenuated renal injury by decreasing NOX2-mediated ROS production and enhancing the expression of antioxidant enzymes such as HO2, PON1, and PON2—highlighting the redox-sensitive and concentration-dependent nature of sulfide signaling in tissue protection [50]. Similar context dependence was noted in a rat model of myocardial ischemia–reperfusion injury, pretreatment with GYY4137 dose-dependently reduced infarct size and preserved cardiac function, while attenuating oxidative stress and ERK1/2 phosphorylation—indicating that suppression of MAPK signaling may mediate the cardioprotective effects of H_2_S in a concentration-sensitive manner [51].

These mechanisms lead to marked effects in certain cell types:

- In endothelial cells, H_2_S promotes angiogenesis by stimulating ERK activation [52].
- It enhances cell proliferation through ERK signaling, although at high concentrations it can induce apoptosis [53].

Thus, hydrogen sulfide finely modulates the MAPK/ERK pathway, exerting biphasic effects depending on the biological context. At low concentrations, it may facilitate proliferation, angiogenesis, and survival, while at high doses or under oxidative stress, it can inhibit prolonged ERK activation, offering protective or even pro-apoptotic effects.

#### 3.2.5. NF-κB

Nuclear factor kappa B (NF-κB) is a transcription factor complex that plays a central role in regulating inflammatory, immune, proliferative, and cell survival responses. Its excessive activation is implicated in various chronic inflammatory, neurodegenerative, cardiovascular diseases, and cancer. Hydrogen sulfide (H_2_S) exerts dual inhibitory activity on NF-κB: both short-term and long-term.

Short-term molecular activity-H_2_S can rapidly inhibit NF-κB activation through several mechanisms, including

- Suppression of IκBα phosphorylation, preventing nuclear translocation of the NF-κB complex (mainly p65/p50) [54].
- S-sulfuration (post-translational modification) of critical cysteine residues in key NF-κB pathway proteins, reducing their activity [54].
- Activation of antioxidant pathways (such as Nrf2), which counteract redox-dependent activation of NF-κB [55,56].

Long-term epigenetic activity-H_2_S also influences the epigenetic regulation of inflammation, leading to:

- Inhibition of histone deacetylases (HDACs), promoting a histone acetylation pattern that represses NF-κB-mediated pro-inflammatory genes [57].
- Suppression of pro-inflammatory cytokines such as IL-1β, IL-6, TNF-α, and cyclooxygenase-2 (COX-2), while enhancing IL-10 production, exerting systemic anti-inflammatory effects [16].
- Induction of microRNAs that negatively regulate components of the NF-κB pathway [58]. At the post-transcriptional level, H_2_S signaling is intricately modulated by non-coding RNAs, particularly microRNAs. Among these, miR-21 has emerged as a key node in inflammatory contexts. Treatment with the slow-releasing donor GYY4137 upregulates miR-21 expression, which in turn activates the Akt pathway and contributes to endothelial protection, reduced apoptosis, and vascular regeneration [59]—mechanisms highly relevant to inflammatory and ischemic conditions that may benefit from balneotherapeutic intervention.
- Indirect epigenetic regulation via activation of sirtuins (SIRT1), which deacetylate the p65 component, thereby inhibiting its transcriptional activity [60,61].

Nevertheless, although H_2_S is known to modulate inflammatory pathways, stimulation of peripheral blood mononuclear cells (PBMCs) with NaHS at concentrations up to 1 mM did not induce NF-κB activation, as measured by p65 phosphorylation and transcriptional activity. This contrasts with earlier reports of synergistic effects when NaHS is combined with LPS, highlighting that sulfide-mediated signaling is highly context-dependent and sensitive to cellular priming conditions [62].

These combined actions position H_2_S as a key immunomodulator with potential applications in the treatment of inflammatory and autoimmune conditions, and in the control of oxidative stress-induced tissue damage.

#### 3.2.6. Epigenetic Modulation (Sirtuins, HDACs, DNMTs)

**Figure 2:**
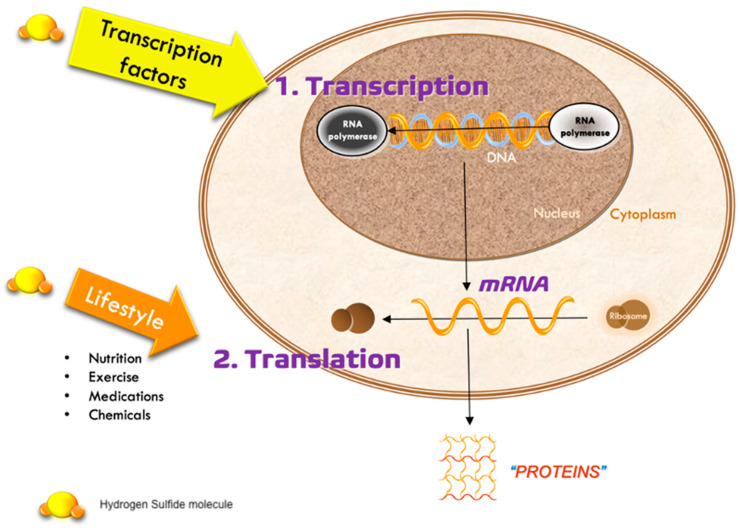
Hydrogen sulfide has a positive or negative influence on the cellular processes of transcription and translation, can have influence the epigenetic cells behavior.

Among the most relevant actions of hydrogen sulfide (H_2_S) in the context of cellular biology is its capacity to modulate epigenetic processes, that is, those which regulate gene expression without altering the DNA sequence Figure 2.

One of the primary epigenetic mechanisms modulated by H_2_S is histone acetylation. H_2_S has been shown to inhibit the activity of histone deacetylases (HDACs), particularly class I and II, which promotes increased histone acetylation and a more relaxed chromatin structure, thereby facilitating gene transcription. This HDAC inhibition has been linked to cytoprotective, anti-inflammatory, and antioxidant effects in various cellular models [63].

In addition, H_2_S participates in direct epigenetic modification via S-sulfuration (persulfidation), affecting both histones and transcription-regulating proteins, including SIRT1. This modification alters the function of transcription factors and chromatin-related enzymes, influencing gene regulation in a redox-dependent manner [35,64].

Another indirect epigenetic pathway modulated by H_2_S is related to sirtuins, particularly SIRT1, a NAD^+^-dependent deacetylase that plays a key role in cellular longevity, metabolic regulation, and stress response. H_2_S can enhance SIRT1 activity by preserving intracellular NAD^+^ levels and improving mitochondrial bioenergetics, thereby contributing to the suppression of inflammation through deacetylation of the NF-κB transcription factor [65,66,67].

Finally, some studies suggest that H_2_S may influence DNA methylation, histone modifications, and the regulation of microRNAs and other non-coding RNAs, possibly through modulation of methyltransferase enzyme activity or by altering the intracellular redox state. However, these effects still require further experimental characterization [63,68,69], Table 4.

**Figure 3:**
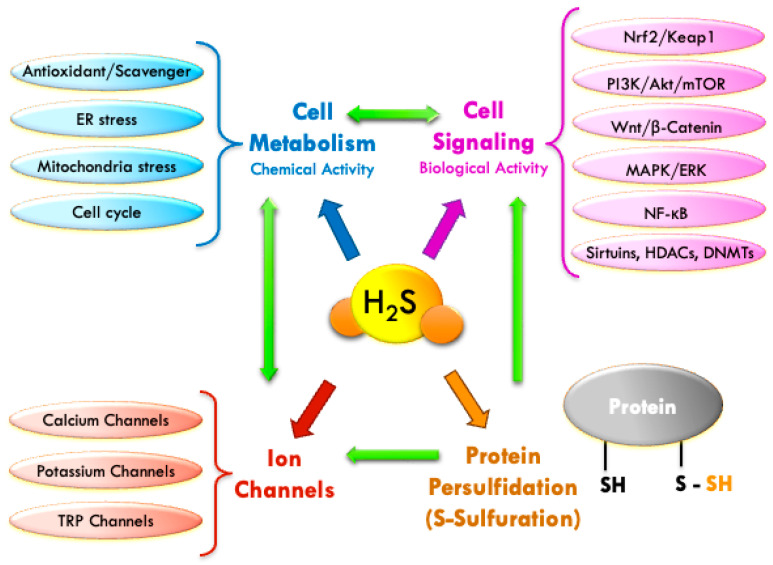
Molecular and cellular targets of hydrogen sulfide (H_2_S). Chemical mechanism on cellular metabolism (blue). Intracellular biological signaling and transcription factors (pink). Protein modifications—persulfidation (orange) and action on ion channels (red). Persulfidation mediates biological activity and ion channels. Similarly, the chemical activity of hydrogen sulfide mediates ion channels, and vice versa. There are cross-relationships between chemical and biological action of hydrogen sulfide. ER: endoplasmic reticulum; -SH: thiol; -SSH: hydropersulfide; TRP: transient receptor potential.

In summary, the ability of H_2_S to modulate various epigenetic marks positions it as a significant epigenetic regulator, with implications in aging, inflammation, tissue repair, and degenerative diseases Figure 3.

## 4. Preclinical and Clinical Evidence

### 4.1. Dermatological Applications in Balneotherapy

Current scientific evidence suggests that balneotherapy has great potential to improve both individual well-being and public health, extending far beyond the conventional treatments offered in spas [70].

Although the biological mechanisms responsible for the benefits of immersion in mineral-medicinal waters and the application of peloids are not fully understood, evidence indicates that neuroendocrine and immunological responses including both humoral and cellular immunity—play a central role in their effectiveness. These responses translate into anti-inflammatory, analgesic, antioxidant, chondroprotective, and anabolic effects, as well as integrated regulation of the neuroendocrine–immune axis in various pathologies [71].

The so-called **“bioregulatory effect of balneotherapy”** has been proposed as a key mechanism of efficacy. This effect consists of reducing systemic pro-inflammatory mediators while preserving an effective innate immune response, ensured by stimulation—or at least the absence of deterioration—of neutrophil-mediated defenses such as phagocytosis and microbicidal activity [72].

Balneotherapy consistently induces modulation of the immune system. After treatment, a significant decrease in the production of pro-inflammatory cytokines such as IL-1β, IL-6, and TNF-α is observed, along with enhanced phagocytic activity of monocytes, reinforcing their ability to eliminate pathogens. This dual effect—reducing inflammation while maintaining or improving innate immunity—highlights a clear bioregulatory action, modulating the immune response without compromising defense against external aggressors [73].

Additionally, the effects of balneotherapy can be partly explained by **hormetic phenomena** associated with nonspecific factors such as heat, which activates the heat-shock response and stimulates the synthesis of heat shock proteins (HSPs). At the same time, specific biochemical factors present in certain waters, such as **hydrogen sulfide (H_2_S)**, contribute to the modulation of oxidative stress and inflammatory pathways.

The use of sulfurous waters containing H_2_S in dermatological diseases is supported by sufficient clinical and preclinical studies. For many years, it has been known that sulfur regulates epidermal differentiation [74,75].

#### 4.1.1. Mechanisms of Action on the Skin

We can divide cutaneous activity into two distinct areas:

1. Effects on Barrier Function and the Cutaneous Microbiome
  H_2_S acts on keratinocytes and fibroblasts, regulating their differentiation, reducing pro-inflammatory cytokines, and activating the SIRT1 pathway with anti-aging effects.
  Clinical improvement has been observed in psoriasis, atopic dermatitis, acne, and rosacea after baths with sulfurous waters, attributed to the synergistic antioxidant, keratolytic/keratoplastic, and bacteriostatic effect [76,77,78].
  In addition, H_2_S and derived molecules have been shown to stimulate the production of type I and III collagen, with implications in dermal regeneration, supporting its use in thermal cosmetics and regenerative aesthetic medicine [79].
  H_2_S improves epidermal barrier function by stimulating keratinocyte proliferation and differentiation, with increased production of structural proteins such as filaggrin, loricrin, and claudins, which are essential for stratum corneum integrity [80].
  It also enhances the synthesis of epidermal lipids (ceramides, cholesterol, free fatty acids), fundamental for cutaneous impermeability [81].
  Moreover, H_2_S exhibits selective antimicrobial activity, inhibiting pathogens such as *Staphylococcus aureus* and *Malassezia* spp., without significantly altering the resident microbiota, which is especially useful in atopic dermatitis, seborrheic dermatitis, and inflammatory acne [82].
2. Antioxidant, Anti-inflammatory, and Anti-aging Activity
  As previously noted, H_2_S has potent antioxidant capacity through **Nrf2 activation**, which increases the expression of HO-1, GPx, and SOD, key players in protection against UV radiation and pollution [83].
  Furthermore, persulfidation of regulatory proteins such as Keap1 modulates adaptive stress responses. It also inhibits NF-κB activation, reducing expression of IL-1β, TNF-α, and COX-2, which are central to inflammatory skin diseases [5].
  Its action on the **SIRT1–FoxO3a pathway** promotes DNA repair, mitochondrial energy regulation, and cellular longevity, consolidating its role as a natural anti-aging agent and decreasing “silent inflammation” [84].
  As a summary, Table 5 lists the clinical cutaneous benefits associated with dermatological treatment based on its mechanism.

#### 4.1.2. Dermatological Diseases Treatable with Sulfurous Waters

Clinical and observational studies support the use of H_2_S-rich sulfurous waters in various dermatological pathologies [85], Table 6:

- **Psoriasis:** Elevated H_2_S concentrations modulate immune responses by reducing Th17/Th1 cytokines (such as IL-8 induced by IL-17/IL-22) and decreasing matrix metalloproteinases (MMP-2, MMP-13). Potential reduction of MMP-9 has also been observed, usually elevated in psoriatic patients, resulting in clinical improvement of erythema, pruritus, and scaling [86,87,88,89].
- **Rosacea:** High-H_2_S waters inhibit NF-κB activation and reduce pro-inflammatory mediators (IL-6, IL-8, TNF-α), attenuating inflammation induced by LL-37 peptide, decreasing angiogenesis, erythema, and *Demodex folliculorum* proliferation [90].
- **Seborrheic dermatitis:** High-concentration sulfurous baths decrease erythema and fungal components (*Malassezia* spp.) without harming resident microbiota [91].
- **Atopic eczema:** Medium–low concentration sulfurous baths restore barrier function and reduce SCORAD index, decreasing *S. aureus* colonization [92,93].
- **Idiopathic or senile pruritus:** H_2_S donors reduce mast cell activation and IL-31 expression in experimental models, suggesting an antipruritic effect [6,94].
- **Wound healing:** H_2_S supplementation accelerates healing through VEGF stimulation and oxidative stress reduction [95].
- **Well-being:** Low concentrations of H_2_S promote keratinocyte proliferation, activate sirtuins and mitochondria, improve microcirculation, and delay cellular aging [10,96,97].

### 4.2. Rheumatological/Locomotor System Applications in Balneotherapy

Hydrogen sulfide (H_2_S) plays a key role in the pathophysiology and treatment of multiple musculoskeletal diseases, including those of rheumatologic, degenerative, and post-traumatic origin. Its therapeutic potential has traditionally been harnessed in balneotherapy through baths in sulfurous waters and, more recently, has been supported by molecular studies and clinical trials that confirm its mechanisms of action and therapeutic benefits [98].

#### 4.2.1. Mechanisms of Action in Osteoarticular Tissues

H_2_S, present in sulfurous mineral-medicinal waters, exerts beneficial effects in rheumatologic and musculoskeletal diseases thanks to its **anti-inflammatory, antioxidant, analgesic, and regenerative actions**, both at the cellular and molecular levels. Balneotherapy has shown good clinical outcomes in chronic musculoskeletal disorders [99] and in joint diseases [100].

One of its main mechanisms is the **inhibition of the NF-κB pathway**, significantly reducing the production of pro-inflammatory cytokines (TNF-α, IL-1β, IL-6) and leukocyte adhesion, while also acting as an anti-catabolic agent. These effects have been demonstrated in animal models of arthritis and in cultures of chondrocytes and synoviocytes [101,102]. Additionally, H_2_S acts as an endogenous regulator of the immune system, modulating both innate and adaptive immunity [103].

In parallel, it activates the **Nrf2/HO-1 antioxidant pathway**, increasing defensive enzymes such as superoxide dismutase (SOD) and glutathione peroxidase, while reducing oxidative stress-induced chondrocyte apoptosis [102].

Clinically, the topical application of sulfurous waters and especially peloids prepared with them contributes to **pain modulation** in osteoarticular conditions. H_2_S shows a **dual role** in inflammatory hypernociception: while endogenous gas can induce pain, exogenous administration exerts analgesic effects through modulation of ion channels (Kv7, K_ATP) and mitochondrial function, providing relief in osteoarthritic pain as shown in preclinical models and human studies [102,104]. It has also demonstrated efficacy in neuropathic pain in mice through Nrf2 pathway activation in vGlut2+ neurons [105].

Slow release of H_2_S reduces edema and inflammation, while endogenous deficiency promotes rheumatoid arthritis pathogenesis by inducing fibroblast-like synoviocyte (FLS) inflammation and accelerating bone and cartilage erosion in collagen-induced arthritis models [106].

Additionally, it prevents cartilage calcification [107], a process accelerated in H_2_S deficiency, as seen in osteoarthritic disease [108]. In the field of **bone regeneration**, sulfurous waters have been shown to stimulate the expression of osteogenic markers (BSP, OC, RUNX-2, OPN) in human bone-derived mesenchymal stromal cells, promoting their differentiation into osteogenic lineages [109].

#### 4.2.2. Musculoskeletal Diseases Treatable with Sulfurous Waters

Extensive studies on the application of hydrogen sulfide and H_2_S donors in osteoporosis, periodontitis, muscle atrophy, ischemia–reperfusion, arthritis, and disc herniation have demonstrated their usefulness [110].

The most common application is through **balneotherapy** (baths, showers, irrigations) or **sulfurous peloids** in localized therapies. The diseases with the strongest therapeutic evidence include Table 7:

- **Osteoarthritis (OA):** Particularly in the knee, hip, and spine. H_2_S contributes to functional improvement, pain reduction, and slowing of structural cartilage deterioration [12]. These effects are attributed to inhibition of pro-inflammatory cytokines (TNF-α, IL-1β), suppression of metalloproteinases (MMP-13), and activation of Nrf2-dependent antioxidant pathways [101,111]. Peloids application is especially indicated in localized conditions [112,113].
- **Rheumatoid Arthritis (RA):** Especially in non-acute phases, H_2_S inhibits fibroblast-like synoviocyte proliferation, reduces IL-6 and MMP-3 production, and blocks NF-κB activation, thereby reducing joint destruction [102]. It also inhibits inflammatory mediators, particularly from T lymphocytes and macrophages [114].
- **Spondyloarthropathies:** Such as ankylosing spondylitis, where H_2_S contributes to axial pain relief and improved mobility due to anti-inflammatory and muscle-relaxant effects. Early validation came from Sukenik’s group [115], with recent studies confirming that simple balneotherapy improves outcomes [116]. Thus, sulfurous balneotherapy and muds are considered safe and effective complementary therapies, with sustained clinical effects lasting up to 12 weeks.
- **Chronic Low Back and Neck Pain:** Whether discogenic or due to muscle contracture, sulfurous waters or hot peloids provide clear benefits by inducing vasodilation, muscle relaxation, and improved local tissue metabolism [12]. Peloid application has shown deep local effects combining heat, sustained H_2_S release, and chemical action on musculoskeletal tissues [117,118,119].
- **Fibromyalgia:** Although evidence remains limited, some studies suggest improvement in widespread pain, sleep quality, and overall well-being, likely via modulation of oxidative stress, neuroinflammatory mediators, and autonomic tone [120].
- **Chronic Tendinopathies:** Such as epicondylitis, plantar fasciitis, rotator cuff tendinitis, and enthesopathies. These respond favorably to balneotherapy and local applications of sulfurous peloids, which improve microcirculation and reduce local inflammation [121].
- **Sarcopenia:** H_2_S protects against skeletal muscle aging through activation of autophagy. A recent study identified that this effect is mediated by H_2_S-induced deubiquitination of AMPKα1 by USP5 (ubiquitin-specific peptidase 5), modulated by S-sulfhydration. This process activates the AMPKα1–ULK1 pathway, essential for autophagy regulation [122].
- **Bone healing after fractures:** In vitro and animal studies suggest H_2_S favors bone consolidation by stimulating osteoblast proliferation and differentiation, promoting mineralization, and modulating the inflammatory microenvironment of the injured site [79].

In all these indications, sulfurous peloids provide additional benefits through sustained H_2_S release, deep thermal effects, and localized action, making them especially effective in focal treatments in rheumatology and physiotherapy [123].

### 4.3. Respiratory/ENT Applications in Balneotherapy

Classically, sulphurous mineral-medicinal waters have been used to treat respiratory tract diseases [3]. The respiratory tract is one of the main absorption routes of H_2_S in balneotherapy, especially through inhalation of vapors or nebulization’s with sulphurous waters via the nasal, tracheal, and bronchial mucosa, due to its involvement in immune regulation, inflammation, mucus secretion, and bronchial tone. The respiratory epithelium shows high expression of H_2_S-sensitive receptors, which enables mucoregulator, anti-inflammatory, bronchodilator, and immunomodulatory effects, useful in chronic inflammatory respiratory diseases.

The respiratory epithelium represents a fundamental route of H_2_S action in balneotherapy, both through direct absorption and through systemic physiological activity. Inhalations of sulphurous mineral-medicinal waters, particularly in the form of aerosol, nasal shower, or spray, have demonstrated efficacy in chronic respiratory diseases [124].

#### 4.3.1. Mechanisms of Action in Respiratory Tract

On the respiratory epithelium, hydrogen sulfide stimulates mucin secretion and improves mucus hydration, acting at several main levels [125]:

- **On respiratory epithelium and ciliary function.** Promotes clearance, increasing production of mucin MUC5AC and enhancing electrolyte secretion, which contributes to thinner, more fluid mucus [126]. Activates key ion channels such as CFTR (cystic fibrosis transmembrane regulator) and Cl^−^ and K^+^ channels, thus restoring mucociliary transport. This mechanism is particularly relevant in diseases characterized by dense mucus, such as cystic fibrosis and chronic bronchitis [127].
  Protects the epithelium against apoptosis induced by oxidative stress and endoplasmic reticulum (ER) stress, mainly through activation of the Nrf2 pathway. This activation enhances synthesis of endogenous antioxidants such as superoxide dismutase (SOD) and catalase, which reduces cell death under conditions of environmental aggression, including exposure to tobacco smoke and atmospheric pollutants [128]. Exerts a potent bronchodilator effect through several complementary mechanisms. Activates ATP-sensitive K^+^ (K_ATP) channels and large-conductance calcium-activated K^+^ (BK_Ca) channels, which reduces excitability and promotes bronchial relaxation [129].
  Inhibits Ca^2+^ influx or release mediated by InsP_3_ receptors, decreasing the frequency and amplitude of Ca^2+^ spikes and attenuating contractility induced by cholinergic agonists such as acetylcholine. It also reduces bronchial constriction caused by histamine and methacholine, showing a clinically relevant effect in bronchial hyperreactivity [130].
- **Regulation of bronchial tone and antiasthma activity.** Activates K_ATP and BK_Ca channels, producing membrane hyperpolarization and bronchial relaxation [131]. As noted above, it inhibits intracellular Ca^2+^ entry in smooth muscle cells, reducing contractility induced by cholinergic agonists. In asthma models, H_2_S has been shown to reduce the bronchoconstrictor response to histamine and methacholine [132].
- **Local immunomodulation in rhinitis, COPD, and pulmonary fibrosis.** Inhibits activation of M1 alveolar macrophages and favours the anti-inflammatory M2 phenotype, reducing chronic inflammation [133]. Decreases expression of IL-6, IL-8, and TNF-α in pulmonary epithelial cells and in bronchoalveolar lavage fluid [134]. In pulmonary fibrosis, H_2_S decreases fibroblast activation and reduces type I collagen synthesis, preventing fibrotic progression [135,136].

#### 4.3.2. ENT and Pulmonary Diseases Treatable with Sulphurous Waters

Therapeutic use of sulphurous waters has demonstrated benefits in several respiratory conditions, with evidence from controlled clinical trials, observational studies, and systematic reviews, supporting their application in clinical practice [137], as seen in Table 8:

- **Allergic and non-allergic rhinitis.** Inhalations with sulphurous waters significantly reduce nasal congestion, sneezing frequency, and Th2 cytokines (especially IL-5), as well as local IgE concentration. A systematic review reported improvements in mucociliary transport and a decrease in nasal epithelial infiltration and inflammation. Although some studies focus on SO_2_ inhalation in animal models, clinical human evidence supports symptom reduction through immune modulation [125,138,139].
- **Chronic bronchitis and mild-to-moderate COPD.** In COPD patients, inhalation with sulphurous waters improves respiratory and clinical parameters increases FEV_1_, reduces sputum volume, improves exercise tolerance, and decreases oxidative stress. A controlled trial showed a significant reduction of oxidative burst and persistent improvement after 12 days of treatment [140]. A systematic review also reported an improvement in quality of life and a reduction of airway oxidation [141].
- **Chronic pharyngitis and laryngitis.** Although there is less direct clinical evidence, sulfurous waters are considered to act as antiseptic, mucoregulatory, and epithelial-regenerating agents in pharyngeal and laryngeal mucosa. General studies in pulmonary hydrotherapy mention beneficial effects on inflammation and epithelial function, without distinguishing precisely these locations, but providing a plausible basis [141].
- **Mild persistent or intermittent asthma.** In mild asthma, inhalation techniques are used as adjuvant therapy to reduce bronchial hyperreactivity. In both human and animal models, improvements in lung function and inflammatory parameters have been observed after inhalation of mineral waters. Recent reviews report reduced inflammation and improved FEV_1_ and reactivity in mild asthma [141].

### 4.4. Activity on the Cardiovascular System

The activity of hydrogen sulfide (H_2_S) on the cardiovascular system has been widely studied over the last decade, becoming established as an endogenous gasotransmitters alongside nitric oxide (NO) and carbon monoxide (CO) [142].

#### 4.4.1. Mechanisms of Action on the Cardiovascular System

Its effects and mechanisms of action are multiple and varied—at the vascular, cardiac, mitochondrial, and epigenetic levels—depending on the concentration and the pathophysiological context, without a homogeneous mechanism of action across different diseases.

Hydrogen sulfide (H_2_S) is now recognized as a key gasotransmitter in cardiovascular physiology and pathophysiology, with vasodilatory, antioxidant, anti-inflammatory, and vascular-remodeling regulatory actions.

First, its hemodynamic action is explained mainly by the opening of ATP-sensitive potassium channels (K_ATP) in vascular smooth muscle, which induces hyperpolarization and relaxation [38,143]. This mechanism is complemented by its interaction with the nitric oxide (NO) system, since eNOS sulfhydration enhances NO bioavailability and amplifies the vasodilatory response [144,145]. These processes underpin its role in controlling blood pressure and peripheral resistance.

In the sphere of endothelial protection and anti-atherogenic effects, H_2_S activates the Nrf2 pathway via persulfidation of Keap1, increasing the expression of defensive enzymes such as HO-1 and NQO1, with antioxidant and anti-inflammatory effects [37,42]. In ischemia–reperfusion models, this activation confers cardioprotection by reducing oxidative damage and preserving mitochondrial function [146]. In addition, sulfhydration of sirtuins (SIRT1 and SIRT2) enhances their deacetylase activity, modulating endothelial senescence and reducing the progression of atherosclerosis [64,84,147].

Another crucial mechanism is inhibition of the NF-κB pathway, both by direct sulfhydration and by epigenetic regulation (HDAC6/MyD88), which reduces transcription of pro-inflammatory cytokines (TNF-α, IL-1β, IL-6) and adhesion molecules, limiting leukocyte recruitment and vascular inflammation [54,57]. This effect has shown relevance for preventing restenosis after angioplasty [58,147].

H_2_S also acts as an antioxidant and bioenergetic modulator. It increases the synthesis of reduced glutathione (GSH) and the activity of antioxidant enzymes such as SOD and catalase, reducing the production of reactive oxygen species [81,148]. At the mitochondrial level, it regulates redox balance and bioenergetic efficiency, acting even as an oxygen sensor under hypoxic conditions [32].

With respect to vascular remodeling and fibrosis, it exerts anti-proliferative and anti-migratory effects on vascular smooth muscle cells (VSMCs), reducing intimal hyperplasia and post-injury remodeling [58]. It also attenuates fibrotic processes mediated by TGF-β and epithelial–mesenchymal transition, protecting against vascular and myocardial fibrosis [47,135]. Notably, at high concentrations it can activate the MAPK pathway and promote apoptosis, highlighting the existence of a dose-dependent therapeutic window [53].

In the context of angiogenesis, a pro-angiogenic role has been described via activation of the Akt pathway and increased VEGF signaling, promoting neovascularization and tissue repair [43]. These effects also contribute to the cardio protection observed in ischemia–reperfusion models [146].

Finally, translational and clinical studies with balneotherapy in sulfurous waters have shown benefits for cutaneous and muscular microcirculation, as well as hemorheological effects, including reduced blood viscosity and improved erythrocyte deformability [12,149]. These findings directly link the molecular mechanisms described to the clinical effects observed in cardiovascular patients treated with sulfurous waters or H_2_S-rich peloids [15,16,19,68].

#### 4.4.2. Cardiovascular Diseases Treatable with Sulfurous Waters

H_2_S is an essential modulator of the cardiovascular system with multiple beneficial effects: vasodilation, protection against ischemic injury, epigenetic modulation, anti-inflammatory activity, and prevention of atherosclerosis. Its potential therapeutic profile makes it an emerging target in current cardiovascular research Table 9:

- Regulation of vascular tone and vasodilation. H_2_S induces potent vasodilation by activating ATP-sensitive potassium channels (K_ATP) in vascular smooth muscle, causing potassium efflux and membrane hyperpolarization, which relaxes the vessel. This effect contributes to lowering blood pressure and maintaining vascular tone [38].
  In addition, H_2_S interacts synergistically with NO by stimulating the expression of endothelial nitric oxide synthase (eNOS) and increasing NO bioavailability, possibly through the formation of hybrid compounds such as nitrosopersulfide [144].
- Cardioprotection in ischemia–reperfusion. In ischemia–reperfusion models, H_2_S exerts mitochondrial and antioxidant protection, reducing the production of reactive oxygen species (ROS) and activating the Nrf2 transcription pathway, with the consequent increase in antioxidant enzymes such as HO-1 and NQO1. At the mitochondrial level, persulfidation of cyclophilin D prevents opening of the mitochondrial permeability transition pore, a key step to avoid cell necrosis [146].
- Effects on the myocardium. H_2_S directly modulates cardiac function. It improves myocardial contractility, reduces post-infarction fibrosis, and contributes to repair of damaged tissue through induction of angiogenesis, mediated by increased expression of VEGF (vascular endothelial growth factor) [150].
- Anti-inflammatory and anti-atherosclerotic action. H_2_S inhibits activation of the NF-κB factor, leading to reduced expression of pro-inflammatory cytokines such as TNF-α, IL-1β, and IL-6, as well as vascular adhesion molecules such as VCAM-1 and ICAM-1. This action decreases endothelial inflammation, leukocyte adhesion, and formation of atherosclerotic plaques. In addition, it reduces LDL oxidation and limits the proliferation of vascular smooth muscle cells, thus slowing atherosclerosis progression [57].
- Blood pressure control. Studies in animal models with deletion of the CSE enzyme have shown that the absence of endogenous H_2_S production is associated with sustained elevation of basal blood pressure, reduced endothelial vasodilation, and increased peripheral vascular resistance, confirming its physiological role as a modulator of hemodynamic balance [143].
- Epigenetic mechanisms and cardiovascular longevity. Hydrogen sulfide (H_2_S) exerts relevant epigenetic effects in the cardiovascular system through post-translational modification of key proteins. One of the best-documented mechanisms is sulfhydration of the p65 subunit of the NF-κB transcription factor, which prevents its activation and nuclear translocation. This epigenetic modification reduces the expression of pro-inflammatory genes such as TNF-α, IL-1β, and IL-6, and diminishes the vascular inflammatory response, contributing to greater endothelial longevity and functionality [54].
  This effect represents a significant epigenetic pathway by which H_2_S protects against chronic vascular damage, regulates the cellular redox state, and contributes to long-term maintenance of hemodynamic balance and endothelial integrity [151].

### 4.5. Activity on Gastrointestinal Mucosa and Related Organs

The gastrointestinal mucosa is continuously exposed to endogenous and exogenous substances with erosive potential, capable of inducing gastric ulcers. In the context of the hydropinic cure with sulfurous waters, most of the ingested hydrogen sulfide (H_2_S) tends to be released as a gas and eliminated by belching. This phenomenon is explained because, in the acidic environment of the stomach (pH ≈ 1–2), the ionized forms of sulfides (HS^−^) quickly gain a proton, becoming molecular H_2_S, which is highly volatile and easily expelled into the oral cavity [10,152].

#### 4.5.1. Mechanisms of Action of the Hydropinic Cure

Nevertheless, due to its lipophilicity and small molecular size, a fraction of the released H_2_S could diffuse through the gastric mucosa and enter the local or systemic circulation.

Upon reaching the duodenum, pH increases significantly due to pancreatic bicarbonate secretion, which favors the conversion of H_2_S into its ionized form (HS^−^), more stable and persistent in the intestinal milieu, where it can act as a modulator at the microbiome–mucosa interface [153]. This stabilized form can continue its transit along the digestive tract, be absorbed distally, or interact with the intestinal microbiota.

Although precise quantitative data in humans are lacking, animal studies have demonstrated intestinal absorption of H_2_S and systemic distribution after oral administration [43]. In addition, the human intestine produces endogenous H_2_S via epithelial enzymes, and exogenous H_2_S via anaerobic sulfate-reducing bacteria (SRB) such as *Desulfovibrio* spp., *Bilophila* spp., or *Fusobacterium* spp., which metabolize sulfur-containing amino acids (cysteine, taurine) and dietary sulfates [20,154].

At physiological concentrations, H_2_S is cytoprotective and anti-inflammatory, whereas in excess it can be toxic for the intestinal epithelium [155]. Solutions of sulfide administered orally or as enemas are absorbed rapidly, although without conclusive quantitative data in humans (U.S. Environmental Protection Agency. Hydrogen sulfide health effects- EPA-600/1-78-018, 1978). Exhaled-air studies have also detected residual H_2_S linked to gastrointestinal phenomena such as diarrhea or bacterial overgrowth, demonstrating its transit from the gut to the lungs via the bloodstream [21].

H_2_S also plays a key role in gut–brain axis communication as an endogenous gasotransmitter and bacterial metabolite. Under physiological conditions it helps maintain the integrity of the intestinal barrier and the blood–brain barrier (BBB). In excess, however, it may promote neuroinflammation and participate in neurodegenerative diseases such as Alzheimer’s or Parkinson’s disease [156,157,158].

In addition, ingested H_2_S is relevant to other associated organs:

- Liver: one of the main H_2_S-producing organs, synthesized by hepatocytes, Kupffer cells, and sinusoidal endothelial cells, enabling autocrine and paracrine functions [151].
- Kidney: promotes renal vasodilation through K_ATP channel opening, regulates glomerular flow, modulates the renin–angiotensin–aldosterone axis, and participates in acid–base homeostasis via tubular ion transport [159].

#### 4.5.2. Diseases of the Gastric Mucosa Treatable with Sulfurous Waters

Ingestion of sulfurous waters exerts an immediate effect on the oral mucosa and, chiefly, on the gastric mucosa. H_2_S at moderate concentrations protects and repairs injured mucosa due to its antioxidant and anti-inflammatory properties [160]. Inhibition of endogenous H_2_S synthesis reduces COX-2 expression and prostaglandin (PGE_2_) production, changes that are reversed when H_2_S is restored [161]. Beneficial effects include [162]:

- Peptic ulcer (gastric and duodenal). H_2_S increases mucosal blood flow, inhibits leukocyte infiltration, and decreases oxidative stress. It favors healing of ulcers induced by gastrotoxic drugs such as NSAIDs. Inhibition of endogenous synthesis reduces prostaglandins (PGE_2_) and COX-2, worsening damage, while H_2_S restitution reverses these effects [161]. H_2_S-NSAID derivatives show lower gastrolesivity while maintaining anti-inflammatory and analgesic efficacy.
- Chronic gastritis (inflammatory and erosive). The anti-inflammatory capacity of H_2_S decreases pro-inflammatory cytokines (IL-1β, TNF-α). It regulates angiogenesis and mucus/bicarbonate secretion, protecting mucosa from injurious agents.
- Gastric lesions due to stress or alcohol. H_2_S reduces oxidative damage and improves gastric microcirculation in experimental models of ethanol- or stress-induced injury. It favors tissue repair through stimulation of angiogenic factors and increased blood flow.
- Functional gastric disorders with acid hypersecretion. By stimulating bicarbonate and prostaglandins, H_2_S helps buffer gastric acidity, protecting mucosa in hyperchlorhydria.

Maintenance of adequate blood flow is essential for mucosal defense and repair, with H_2_S-mediated vasodilation as a central mechanism.

#### 4.5.3. Intestinal Diseases Treatable with Sulfurous Waters

H_2_S is a key microbial metabolite with dual effects on intestinal and systemic health. Its role is ambivalent. In excess, usually associated with dysbiosis or proliferation of sulfate-reducing bacteria, it can damage the intestinal barrier, degrade protective mucins, increase permeability (leaky gut), and facilitate translocation of pro-inflammatory molecules into the systemic circulation [156]. These mechanisms promote chronic inflammation and deterioration of intestinal function [20].

The intestinal microbiota not only produces H_2_S but can also metabolize it. Some species express enzymes such as sulfide:quinone oxidoreductase and persulfide dioxygenase, capable of degrading or transforming H_2_S into less toxic compounds, contributing to local gas balance [163].

This balance is also essential for central nervous system health via the gut–brain axis. A physiological level of H_2_S preserves epithelial integrity and exerts systemic anti-inflammatory effects, whereas overproduction compromises both this barrier and the BBB, activating neuroinflammatory processes related to diseases such as Alzheimer’s and Parkinson’s [156].

Recent research shows that in small intestinal bacterial overgrowth (SIBO, ≥1000 CFU/mL on MacConkey agar) there is an excess of hydrogen and H_2_S. This condition associates with marked microbiota disturbance, with considerable increases in *Escherichia coli* and *Klebsiella* spp. and a notable drop in common species diversity and abundance. These changes increase fermentative capacity, H_2_/H_2_S production, and biogenic amine synthesis, contributing to abnormal gastrointestinal symptoms and metabolic dysfunction [164].

Conclusion, intestinal H_2_S is a double-edged mediator. Beneficial in balance since it maintains epithelial integrity, regulates inflammation, and stabilizes biological barriers (intestinal and cerebral). Harmful in excess by compromising mucosal integrity, promoting local and systemic inflammation, and negatively impacting neuroimmunity.

The balance depends on factors such as microbiota composition, diet, and health status. Understanding these mechanisms opens the way to therapeutic strategies based on probiotics, prebiotics, parabiotics, postbiotics, or dietary interventions aimed at modulating the microbiota and maintaining adequate H_2_S levels.

It should always be borne in mind that the contribution of H_2_S from the hydropinic cure appears to be limited compared with endogenous microbial production.

#### 4.5.4. Liver Diseases Treatable with Sulfurous Waters

The liver is the principal metabolic center of the body, essential for the regulation of glucose, lipids, detoxification, and antioxidant defense. In this organ, hydrogen sulfide (H_2_S) is not a mere byproduct but an endogenously produced signaling molecule capable of modulating functions critical to hepatic homeostasis [165].

H_2_S plays a relevant role in redox homeostasis and hepatic detoxification processes through activation of the Nrf2/ARE pathway. This signaling stimulates the expression of antioxidant genes such as HO-1, NQO1, GST, GCLM, GCLC, SOD, and catalase, which confers protection to hepatocytes against oxidative stress and the action of xenobiotics. In addition, H_2_S regulates genes involved in fatty-acid β-oxidation, gluconeogenesis, and lipogenesis, helping prevent hepatic steatosis and other metabolic disorders. Another effect is vasodilation in hepatic sinusoids, mediated by the opening of K_ATP channels, which improves portal perfusion and favors overall liver function [165].

In animal models, exogenous administration of H_2_S has shown hepatoprotective effects: in obese mice fed high-fat diets it reduced the accumulation of triglycerides and cholesterol, inhibited the expression of fatty acid synthase (FAS), and stimulated carnitine palmitoyltransferase 1 (CPT1), key in β-oxidation. In parallel, it enhanced antioxidant defenses by increasing the activity of enzymes such as superoxide dismutase (SOD) and glutathione peroxidase (GPx), reducing levels of lipid peroxidation [166].

In situations of liver injury or fibrosis, H_2_S exerts beneficial effects by modulating oxidative stress, inflammation, autophagy, and glucolipid metabolism, which makes it a key mediator in chronic liver pathologies, including chronic alcoholism. Although its role in diabetes is not yet fully understood, experimental evidence supports its influence on the regulation of glycemic homeostasis [167].

The hydropinic cure with sulfurous waters has been associated with reductions in blood levels of glucose and oxygen, as well as improvements in quality of life (SF-36). H_2_S also appears to be involved in the regulation of endoplasmic reticulum stress, a relevant aspect in metabolic diseases such as diabetes [168].

From a metabolic standpoint, adequate endogenous production of H_2_S favors the regulation of energy metabolism, contributing to the prevention of conditions such as obesity or metabolic syndrome. The gas participates in cellular pathways involved in glucose control, modulating inflammatory and mitochondrial factors and essential metabolic adaptations [169,170].

Nutrition exerts a modulatory role in hepatic H_2_S synthesis. In animal models, fructose consumption, in contrast to glucose, has been observed to significantly reduce hepatic H_2_S production, especially during gestation. This decrease is associated with increased oxidative stress, dyslipidemia, and hepatic steatosis, conditions linked to the development of insulin resistance and alterations in carbohydrate metabolism [171].

Deficiency in endogenous H_2_S production has been associated with the progression of severe liver disease, while administration of exogenous donors has shown protective effects against hepatic dysfunction. However, its role is not univocal: recent studies indicate that H_2_S can exert both protective and deleterious functions in the liver, depending on the pathophysiological context. Determining factors include the type of pathology, endogenous H_2_S levels, the dose of donors administered, and the duration of treatment. In certain clinical situations—such as some liver cancers or acute liver injury—both inhibition of its internal synthesis and exogenous administration can be beneficial, always within a controlled therapeutic framework [172].

In this field, some liver diseases that H_2_S may improve or protect are the following:

- Hepatic steatosis (non-alcoholic fatty liver disease, NAFLD). H_2_S reduces the accumulation of triglycerides and cholesterol. It inhibits lipogenic enzymes (e.g., FAS) and activates β-oxidation (CPT1). It decreases oxidative stress by enhancing SOD and GPx, reducing lipid peroxidation [166]. It also protects against oxidative stress and apoptosis in alcohol-induced liver injury. It favors cellular repair and reduces inflammation in chronic alcoholism.
- Hepatic fibrosis. It modulates oxidative stress and chronic inflammation. It regulates autophagy and glucolipid metabolism. It favors hepatic perfusion through sinusoidal vasodilation [165].
- Diabetes and associated hepatic dysfunction. It contributes to glycemic control by modulating gluconeogenesis and glycolysis. It participates in the reduction of endoplasmic reticulum stress in hepatocytes [167,168,173].
- Metabolic syndrome and obesity. Adequate endogenous production of H_2_S improves energy metabolism and regulates inflammation, mitochondrial function, and metabolic adaptation pathways [169,170].
- General hepatic oxidative stress. Activation of the Nrf2/ARE pathway, which stimulates antioxidant genes (HO-1, NQO1, GCLC), provides defense against xenobiotics and hepatotoxic agents [165].

In severe diseases such as hepatocellular carcinoma or acute liver injury, its role is context-dependent, and both its inhibition and exogenous administration may be useful.

### 4.6. Kidney Diseases Treatable with Sulfurous Waters

Hydrogen sulfide (H_2_S) is recognized as a multifaceted renal gasotransmitter, with essential roles in renal physiology and in defense against injury. At the physiological level, it participates in the regulation of glomerular filtration, tubular sodium handling, blood pressure control, and cellular energy production. From a pathological standpoint, several studies have demonstrated its protective effect in diabetic nephropathy [174], in renal fibrosis [175], as well as in chronic kidney disease and acute kidney injury [176]. In addition, H_2_S promotes recovery and viability in the context of kidney transplantation thanks to its antioxidant, anti-inflammatory, and cytoprotective properties [177].

These findings position H_2_S as a promising therapeutic target in nephrology, although its clinical use requires caution to avoid undesirable effects arising from dosing or the pathophysiological context. In this sense, sulfurous waters could constitute a useful adjuvant in the management of diabetic nephropathy, renal fibrosis, acute and chronic kidney disease, as well as in the secondary prevention of hyperuricemia and uric acid urolithiasis.

Table 10 summarizes the main possible activities of hydrogen sulfide on the gastrointestinal tract, liver and kidney problems.

### 4.7. Other Indications

We do not wish to conclude this section without underscoring the role of hydrogen sulfide (H_2_S) in other health domains.

- In rehabilitation, recent studies identify H_2_S as a pain modulator via activation/inhibition of TRPA1/TRPV1 and K_ATP channels, exhibiting pro- or antinociceptive effects depending on dose, chemical species, and the inflammatory context [178]. It also enhances micro perfusion through endothelium-dependent vasodilation and augmentation of the NO/cGMP pathway, which is relevant for tissue recovery and therapeutic exercise [15]. Moreover, H_2_S regulates cellular metabolism, autophagy, and homeostasis, with antifibrotic potential and supportive effects on muscle function and aging [122]. In selected clinical settings, benefits on pain and function have been observed in osteoarthritis, and when combined with exercise these improvements may be prolonged [123,179,180,181].
- In psychological and neurological disorders, H_2_S acts as a gaseous neurotransmitter synthesized by CBS, CSE, and 3-MST within the central nervous system, modulating NMDA receptors, K_ATP channels, microglial activity, and the GABA/glutamate balance [63,70,182,183]. Slow-releasing donors such as GYY4137 attenuate neuroinflammation, preserve blood–brain barrier integrity, and improve cognitive performance in animal models [184]. Along the gut–brain axis, endogenous and microbiota-derived H_2_S from sulfate-reducing bacteria influences intestinal permeability, immune signaling, and vagal tone, thereby linking dysbiosis to neuropsychiatric phenotypes [156].

## 5. Routes of Administration and Bioaccessibility of H_2_S

Hydrogen sulfide (H_2_S) exerts biological effects modulated by both its concentration and its release profile. It is not only a quantitative difference, that is, a higher or lower concentration of gaseous H_2_S, but also a qualitative one, since the mechanisms of action vary according to the mode of release. The physiological response differs markedly if H_2_S is released rapidly and explosively, or gradually and in a sustained manner, especially in complex, tightly regulated processes. Therefore, neither the delivery vehicle nor the route of administration has to be uniform for all pathologies [185].

### 5.1. Topical Route: Waters and Peloids

In the context of bathing in sulfurous waters, transdermal absorption of H_2_S is a well-documented phenomenon. The molecular fraction of H_2_S, being lipophilic, diffuses passively through the stratum corneum and also penetrates via appendageal structures such as hair follicles and sweat glands. In contrast, the hydrosulfide ion (HS^−^), due to its negative charge, crosses the skin barrier with difficulty and shows limited dermal and systemic bioavailability [10].

Experimental studies in animal models and human in vitro systems have confirmed that intact skin is an effective barrier against brief exposures, even at high H_2_S concentrations. However, under balneotherapy conditions—warm water, prolonged exposure, high humidity, large body surface areas, and occasional occlusion—cutaneous absorption of H_2_S increases significantly. These conditions favor the opening of porous channels, increase cutaneous blood flow, and raise gas solubility in the epidermis and dermis. Once absorbed, H_2_S can exert local and low-intensity systemic effects without reaching toxic cumulative levels [185].

H_2_S acts in synergy with other components of mineral-medicinal waters, such as magnesium, sodium, sulfates, bicarbonate, carbon dioxide, or trace elements [186,187]. The combination of these minerals enhances vasodilation, improves epidermal hydration, and favors the transdermal absorption of the gas [71]. In particular, CO_2_, including in bicarbonated waters, stimulates tissue oxygenation and perfusion through activation of endothelial nitric oxide synthase (eNOS), creating a biochemical environment favorable to the effects of H_2_S [188].

The effective concentration of H_2_S in sulfurous waters can be reduced in a controlled manner, a relevant aspect in spa practice. The presence of oxygen (aeration, bubbling) and an increase in temperature promote its oxidation and volatilization, transforming H_2_S into less active species such as thiosulfate (S_2_O_3_^2−^) and sulfate (SO_4_^2−^) [10].

The most usual temperature range in dermatologic and rheumatologic indications is 34–38 °C. Low temperatures (<34 °C) reduce H_2_S volatilization and are useful in scaly dermatoses or in patients with cardiovascular intolerance. High temperatures (>38 °C) increase gaseous release and vasodilation, although with greater hemodynamic load. The choice must be individualized according to pathology and clinical status.

The optimal therapeutic immersion time ranges from 10 to 20 min. Longer exposures increase the risk of hypotension, fatigue, and headache due to H_2_S inhalation. In patients with heart disease, it is advisable to start with short sessions (8–10 min), increasing progressively. Classical protocols include 3–6 sessions per week for cycles of 2–3 weeks, adjusting frequency and duration to tolerance and clinical response.

During bathing, H_2_S penetrates simultaneously via the cutaneous and respiratory routes. While transdermal absorption occurs progressively, the inhalation route allows faster passage of the gas through the alveoli into the systemic circulation. This generates a dual bioavailability profile: rapid via inhalation and sustained via the skin, which enhances therapeutic effects in systemic diseases such as rheumatologic, cardiovascular, or metabolic conditions.

Sulfurous peloids are homogeneous mixtures of sulfurous mineral water with organic and inorganic solid components (clays, silts, peats, algae, plant remains) that have matured under controlled conditions to form a poultice. This process fixes sulfides, minerals, and bioactive compounds in the solid matrix [189,190].

General application of peloids has been associated with a significant increase in systemic cortisol levels, together with a marked reduction in IL-8 concentration and greater phagocytic and microbicidal activity by neutrophils. Taken together, these effects reflect the induction of immuno-neuro-endocrine stabilization, considered one of the mechanisms underlying the clinical benefits observed in spa intervention [191].

Sulfurous peloids also provide sustained release of molecular H_2_S. Applied to the skin they act as reservoirs, releasing the gas slowly and prolonging contact time. This avoids excessive concentration and maintains a stable therapeutic effect, particularly useful in chronic dermatologic diseases (psoriasis, dermatitis, eczema) and in localized musculoskeletal injuries (osteoarthritis, tendinopathies, myalgias) [185].

The usual application temperature ranges from 38 to 46 °C, adjusted according to indication and patient tolerance. In general, increasing temperature appears to influence the efficacy of the technique [192]. Heat favors the release and diffusion of H_2_S, increases vasodilation and perfusion, and produces a deep thermal effect in muscles and joints, reducing spasm, stiffness, and pain [193,194].

In addition, peloids supply minerals and trace elements (calcium, magnesium, sodium, potassium, silica, etc.), with potential remineralizing, anti-inflammatory, and wound-healing effects. The occlusion they generate increases local humidity and enhances cutaneous penetration of these elements [195].

Taken together, sulfurous peloids integrate three main actions: a deep thermal effect, mineral supply, and sustained release of H_2_S. This makes them a highly effective balneotherapeutic tool for both local treatments and for rehabilitation and dermatology programs [151], as seen in Table 11.

### 5.2. Respiratory Route

Thermal techniques with sulfurous waters for respiratory conditions are an effective and safe complementary treatment for various chronic otorhinolaryngological and pulmonary diseases [196,197]. The antiseptic, anti-inflammatory, mucolytic, antioxidant, and regenerating properties of H_2_S in these waters are well documented, and their action depends not only on the chemical composition of the water but also on the dosage form, pH, temperature, and exposure time. They show clear clinical usefulness in rhinitis, sinusitis, pharyngitis, laryngitis, chronic bronchitis, mild asthma, and early COPD.

Application techniques through the respiratory tract with sulfurous waters, as with other kinds of thermal waters, are divided into local techniques and true inhalation techniques. The choice depends on the desired depth of action on the respiratory system.

#### 5.2.1. Local Techniques

Defined as therapeutic procedures intended to bring sulfurous water into direct contact with the mucosa of the upper airways, with a mainly topical objective. Their aim is to exert an immediate effect on inflamed or altered mucosa, acting as antiseptic, anti-inflammatory, and regenerating agents.

The most commonly used modalities are gargles, applied to the oropharyngeal and laryngeal mucosa, and nasal sprays or showers that project large liquid particles (>20 μm), retained in the nasal cavity and oropharynx without penetrating the lower airways. These techniques are especially indicated for ENT conditions such as chronic pharyngitis and laryngitis, allergic and non-allergic rhinitis, and chronic sinusitis.

Their main advantage is localized, direct action, allowing targeted treatment of diseased mucosa without systemic absorption, with good clinical tolerability and few adverse effects.

#### 5.2.2. Inhalation Techniques

Defined as those in which sulfurous water, transformed into vapor or aerosol, is actively inhaled to reach deeper regions of the respiratory tree. Their goal is to combine a topical action on the mucosa with functional effects on pulmonary ventilation. Main modalities:

- Dry inhalations: breathing the gas or vapor released directly from the mineral-medicinal water, without entrained liquid droplets, generating fine particles of approximately 10–20 μm that can deposit in upper and middle airways; useful in rhinitis or early chronic bronchitis.
- Wet inhalations: a mixture of vapor and larger aqueous particles, 20–50 μm, with soothing and mucoregulatory action, primarily on upper airways.
- Nebulization: a very fine and abundant form of wet inhalation producing much smaller particles, 1–5 μm, which can reach bronchioles and alveoli. Indicated in mild asthma, early COPD, or chronic bronchitis.
- Atmiatric techniques: such as steam baths in a cabin, providing diffuse inhalation of particles of heterogeneous size, usually >50 μm, acting mainly on upper and middle airways and adding a beneficial thermal effect.

Advantages include greater reach and depth, allowing not only improved fluidization of secretions and reduced inflammation but also influence on functional parameters such as ventilatory capacity and exercise tolerance.

In summary, whereas local techniques act in a focused manner on nasal, oropharyngeal, and laryngeal mucosa, inhalation techniques extend their range to the lower airways, combining broader therapeutic action with clinical benefits that are both local and modestly functional.

For all these indications, and to ensure mucosal tolerance and avoid irritative reactions, sulfurous water should have a pH close to physiological, a temperature between 35 and 37 °C, and sessions should not exceed 15–20 min. The gaseous bioavailability of H_2_S is a key factor in therapeutic efficacy, so waters with an effective concentration of the gas should be prioritized.

Facilities must have active ventilation to keep H_2_S concentrations < 10 ppm in air (safe occupational limit) by using extraction or air-renewal systems and avoiding airtight covers that accumulate gas.

Sessions are usually 10–20 min daily, in 2–3 week courses. Effects are cumulative, which justifies continued spa therapy.

In sulfurous waters, unlike other mineral-medicinal waters, H_2_S exerts mucoregulatory, keratolytic, antioxidant, and anti-inflammatory action, which explains its particular interest in chronic ENT and bronchopulmonary disorders.

### 5.3. Hydropinic Cure

The hydropinic cure with sulfurous mineral-medicinal waters consists of the controlled ingestion of waters rich in hydrogen sulfide in order to exert therapeutic effects on the digestive tract, metabolism, and the hepatobiliary and renal systems, with possible influence on general physiology.

Although this route has traditionally been used to treat digestive disorders, its effects are now recognized as much broader. Ingested H_2_S can act on the gastric mucosa, influence liver and kidney function, modulate systemic epigenetic signaling, and even, though more limitedly, modify the composition and activity of the intestinal microbiota.

In theory, many processes could benefit from this therapeutic modality, since H_2_S can affect multiple tissues, organs, and systems. Although not widely used in spa practice, experimental animal studies support further research in humans [198]. Numerous everyday foods considered healthy are also known to exert part of their effects through modulation of endogenous H_2_S levels [199].

Effects of the hydropinic cure may be early or delayed, with local and direct actions on the digestive tract or systemic actions, depending on factors such as ingested volume; water temperature; osmotic pressure; and mineral composition and H_2_S concentration.

In practice, administration is progressive, adjusting the dose according to individual tolerance and the actual H_2_S content (mg/L). The usual regimen ranges from 0.6 to 1.5 L per day, always under medical supervision. The higher the concentration of sulfides and accompanying salts, the lower the total volume to be ingested. Very H_2_S-rich waters may cause sulfur burps, nausea, or digestive discomfort, so it is advisable to start with small volumes and increase progressively.

A practical initiation scheme is to start with 200 mL per day, divided into several doses, until the prescribed dose is reached. The first intake is recommended on an empty stomach, divided into three small glasses given at intervals of about 10 min. The second intake is usually before the main meal, although in some cases a three-dose regimen may be prescribed.

The standard spa course lasts 2 to 3 weeks and may be repeated once or twice a year, depending on clinical evolution and therapeutic indications.

## 6. Safety Considerations

Exposure to H_2_S is a potentially serious health risk and requires strict environmental control. Adverse effects depend on both ambient concentration and duration of exposure, with clear differences between prolonged and short-term exposures.

For prolonged exposures, concentrations between 2 and 5 ppm are associated with early symptoms such as nausea, headaches, and eye tearing. The time-weighted exposure threshold limit value has been set at 10 ppm for 8 h days and 40 h weeks, corresponding to the maximum level considered safe for continuous occupational environments ACGIH (American Conference of Governmental Industrial Hygienists, Sharonville, OH, USA); OSHA (Occupational Safety and Health Administration, Washington, DC, USA). Exceeding this limit may progressively increase adverse effects; at 20 ppm fatigue, persistent headache, irritability, dizziness, and memory impairment are common.

For brief exposures, critical values are even more restrictive. A limit of 15–20 ppm has been set as the maximum permissible concentration during 15 min periods per 8 h shift (NIOSH-National Institute for Occupational Safety and Health; EU Directive 2000/39/EC and update, Washington, DC, USA). Levels between 50 and 100 ppm cause acute respiratory irritation, while concentrations of 100–150 ppm are considered immediately dangerous to life or health (IDLH: Immediately Dangerous to Life or Health). Inhalation of 300–500 ppm can induce pulmonary edema, and exposures above 500 ppm are lethal within minutes.

Regulatory evidence highlights the need for continuous environmental monitoring, effective ventilation systems, and, where appropriate, the use of personal protective equipment with supplied air to prevent poisoning. In thermal or spa settings, although concentrations are usually much lower than in industry, adopting occupational safety protocols based on these standards is essential to protect both professionals and users.

Devices exist to measure hydrogen sulfide in the air of spa environments. There are two types: colorimetric tubes and personal portable monitors (detectors).

Colorimetric tubes operate by a color change of a chemical reagent on contact with H_2_S. They are portable, inexpensive, quick to use, and highly sensitive, from 0.2 ppm to several hundred ppm. Their limitation is spot, not continuous, measurement.

Personal portable monitors are generally used by workers in very high-risk environments and are not usual in spa settings. They incorporate electrochemical sensors specific for H_2_S that trigger audible, visual, and vibratory alarms when preset thresholds are exceeded (e.g., 5 ppm, 10 ppm). They have long autonomy, 1–2 years depending on the model, and the advantage of continuous operation Table 12.

## 7. Conclusions

Hydrogen sulfide (H_2_S) has been established in recent decades as a true gasotransmitter with functions comparable to nitric oxide and carbon monoxide. Biochemical, molecular, and clinical evidence positions it as a key molecule in the regulation of cellular homeostasis, redox balance, inflammation and silent inflammation, and epigenetic signaling. Its ability to act in a dual manner—through immediate effects at the chemical level and longer-lasting modulations via epigenetic processes—makes H_2_S a molecule of physiological and therapeutic interest.

From the balneotherapeutic perspective, sulfurous mineral-medicinal waters represent a natural and accessible source of H_2_S whose therapeutic use has been supported by experimental and clinical studies. When properly managed from a hydrochemical and technical standpoint, these waters preserve the bioavailable fraction of the gas and ensure its therapeutic action. Their application in dermatology, rheumatology, and respiratory disease offers benefits ranging from restoration of skin barrier function, regulation of the microbiota, and tissue repair to reduction of osteoarticular inflammation and modulation of bronchial hyperreactivity.

The molecular mechanisms described in this review—including activation of the Nrf2/Keap1 pathway, inhibition of NF-κB, persulfidation of key proteins, activation of ion channels, and modulation of HDACs and sirtuins—provide a solid physiological basis that explains the clinical effects observed. This knowledge has allowed us to transcend the classic empiricism of balneotherapy, providing it with a rigorous scientific foundation that legitimizes its use within integrative medicine and modern pharmacology.

Likewise, the epigenetic activity of H_2_S, with implications for aging, cell repair, and metabolic regulation, opens new research avenues toward its application in regenerative medicine, oncology, neuroprotection, and healthy longevity. In the same way, the development of controlled-release pharmacological H_2_S donors and the incorporation of cosmetic strategies based on this gasotransmitter broaden its therapeutic horizons beyond the spa setting.

In conclusion, H_2_S should be understood as a hinge molecule between redox biology, cellular signaling, and epigenetics, with notable translational potential in various areas of medicine and cosmetics. Balneotherapy with sulfurous waters is not only a historical therapeutic heritage but also emerges as a contemporary and future tool for the prevention and treatment of multiple pathologies, provided that clinical protocols grounded in current scientific knowledge are applied. However, several limitations should be considered. The heterogeneity of sulfurous waters introduces multiple variables—H_2_S/HS^−^ equilibrium, pH, temperature, aeration, accompanying ions, polysulfides, and other gases—that can modify bioavailability. Effective H_2_S doses at the site of action and the impact of different routes of administration during therapeutic applications are seldom known. Further work is needed to characterize these singularities in order to establish more specific protocols for sulfurous waters and to facilitate their translation to clinical practice.

## Figures and Tables

**Table 1:** Range of action of hydrogen sulfide and its ions as a function of pH, temperature and presence of oxygen.

Variable (Range)	Predominant Chemical Species	Bioavailable Fraction	Main Absorption Route in Spa Practice	Therapeutic Implication
**pH** **4.5** **–6.5 (acidic)**	H_2_S (gas)	High (lipophilic)	Topical (diffusion through skin)	Favorable for dermatological applications
**Physio pH** **7.2** **–7.4**	HS^−^ + H_2_S (≈4:1)	Moderate	Inhalation (alveolar uptake of H_2_S gas)	Useful for respiratory indications; monitor exposure limits
**Alkaline pH > 8.0**	HS^−^ ≫ H_2_S	Low (ionized)	Limited	Reduced activity
**Tª < 30 ** **°C**	Higher solubility of H_2_S in water	Moderate	Topical (slow volatilization)	Longer bath retention; mild inhalation
**Tª 30** **–40 ** **°C**	Equilibrium shift to gas phase	High near surface	Combined topical + inhalation	Optimal spa range; increases systemic delivery
**Tª > 40 ** **°C**	Rapid H_2_S volatilization	Variable (declines in water)	Predominantly inhalation (short exposure)	Requires ventilation to avoid toxic peaks
**Low O_2_ (<2 mg L** **^−^** **^1^)**	H_2_S preserved, minimal oxidation	High	Topical/inhalation (stable gas)	Maximisers therapeutic fraction
**High O_2_ (>6 mg L** **^−^** **^1^)**	Oxidation to thiosulfate/sulfate	Very low	Negligible	Loss of activity; avoid aeration

**Table 2:** Synthesis of hydrogen sulfide in different organs [17].

Organ/Tissue	Dominant Enzyme	Functional Relevance
**Brain**	CBS	Neuroprotection, neurogenesis
**Heart/Vessels**	CSE	Vasodilation, blood pressure regulation
**Liver/Kidney**	CBS/CSE	Redox metabolism, fibrosis protection
**Mitochondria (various)**	3-MST	Energy homeostasis, cellular bioenergetics

**Table 3:** Antioxidant mechanisms mediated by H_2_S.

Mechanism	H_2_S Action	Reference
**Direct neutralization**	Scavenging of H_2_O_2_ and •OH	Kimura, 2015 [2]
**Antioxidant enzymes**	↑ SOD, GPx, GSH	Paul, 2015 [4]
**Nrf2 activation**	Genetic transcription of HO-1, NQO1	Yang et al., 2013 [37]
**Persulfidation**	Protein protection	Mustafa et al., 2009 [35]
**Mitochondrial redox**	↑ mitochondrial efficiency ↓ ROS production	Kabil & Banerjee, 2010 [8]

**Table 4:** Summary of molecular targets and physiological effects of H_2_S.

Mechanism	Cellular Target	Physiological Consequence	Therapeutic Implication
**Persulfidation (S-sulfuration)**	Proteins with free cysteine groups	Enzymatic and structural modulation	Cytoprotection, metabolic regulation
**Nrf2 activation**	ARE (Antioxidant Response Elements) in cell nucleus	Induction of endogenous antioxidants	Anti-aging, oxidative defense
**Inhibition of HDACs and DNMTs**	Epigenetic enzymes	Re-expression of silenced genes	Skin repair, longevity
**Activation of sirtuins**	SIRT1/SIRT3 in nucleus and mitochondria	Energy regulation and cellular repair	Epigenetics, tissue protection
**Activation of K_ATP channels**	Cell membrane	Membrane hyperpolarization and vasodilation	Muscle relaxation, analgesia, peripheral circulation
**Inhibition of NADPH oxidase**	Inflammatory cells	Reduction of ROS and free radicals	Anti-inflammatory, neuroprotection
**NF-κB modulation**	Pro-inflammatory pathway	Decrease in pro-inflammatory cytokines	Immunomodulation, pain treatment

**Table 5:** Summary of molecular mechanism and clinical benefit of H_2_S in dermatologic processes.

Effect	Molecular Mechanism	Clinical Benefit
**Antioxidant**	Nrf2 activation; persulfidation; inhibition of NADPHox	Protection against photoaging and oxidative stress
**Anti-inflammatory**	NF-κB inhibition; ↓ IL-1β, TNF-α	Relief of pruritus, psoriasis, eczema
**Epigenetic repair**	Activation of SIRT1/SIR2/SIRT3; inhibition of HDACs & DNMTs	Skin regeneration, cellular longevity
**Microbiome modulation**	Selective action against pathogens	Reduction of dysbiosis in dermatitis and seborrhea
**Barrier restoration**	Stimulation of filaggrin, loricrin, and lipids	Re-epithelialization, hydration, and barrier repair
**Angiogenesis & tissue** **repair**	VEGF activation and fibroblast migration	Healing of ulcers, wounds, and chronic erosions

**Table 6:** Summary of the main dermatological effects of H_2_S, their scientific evidence and the possible mechanism.

Condition	Main Effects	Mechanisms	Evidence
**Psoriasis**	↓ Inflammation, ↓ MMPs	Keratolysis; Th1/Th17 cytokine inhibition; ↓ MMP-9; normalized proliferation	Clinical + preclinical
**Atopic Dermatitis**	↓ *S. aureus*; barrier restoration	Keratoplasia; microbiota rebalancing; lipid improvement; ↓ IL-4/IL-13	Clinical + spa use
**Seborrheic Dermatitis**	↓ *Malassezia* spp.; ↓ erythema	Selective antifungal; localized anti-inflammatory	Clinical-observational
**Rosacea**	↓ LL-37; ↓ erythema; ↓ *Demodex*	Neurovascular inhibition; local immune modulation	Preclinical + empirical
**Inflammatory Acne**	↓ *C. acnes*	Seboregulation; keratolysis; antimicrobial & lipid regulation	Case reports + observational
**Chronic Eczema**	Barrier restoration; ↓ microbial colonization	Lipid improvement; ↑ filaggrin & loricrin	Clinical experience + studies
**Pruritus**	↓ IL-31; ↓ mast cell activation	Neuroimmune modulation; ↓ pruritogenic cytokines	Preclinical + clinical
**Wound Healing**	↑ VEGF; ↓ oxidative stress	Angiogenesis & fibroblast migration	Preclinical + observational
**Well-being**	Improved microcirculation; anti-aging	Sirtuin & NO pathway activation	In vitro + clinical

**Table 7:** Summary of the main Musculoskeletal Diseases treated with H2S, their scientific evidence and the possible mechanism.

Condition	Main Effects	Mechanisms	Evidence
**Osteoarthritis**	↓ Inflammation, ↓ oxidative stress, ↓ apoptosis, ↓ pain	Inhibits NF-κB/MAPK/PI3K; activates Nrf2/HO-1/K^+^ channels	Preclinical + balneotherapy
**Rheumatoid Arthritis**	↓ Synovial inflammation, ↓ FLS proliferation, ↓ erosion	Inhibits cytokines & NF-κB/MAPK; restores H_2_S via nano-carriers	Preclinical + biomarker data
**Skeletal Muscle Aging** **(Sarcopenia)**	↑ Autophagy, ↓ muscle atrophy markers	USP5-mediated AMPKα1 deubiquitination; activation of AMPKα1–ULK1	Preclinical in vitro & in vivo
**Spondyloarthropathies**	↓ Axial pain, ↑ mobility	Anti-inflammatory & myorelaxant effects	Clinical + observational
**Chronic Low Back Pain**	↓ Pain, ↑ local metabolism	Vasodilation, heat effect, H_2_S release	Clinical
**Fibromyalgia**	↓ Generalized pain, ↑ sleep, ↑ well-being	Oxidative stress & neuroinflammation modulation	Observational
**Chronic Tendinopathies**	↓ Inflammation, ↑ microcirculation	Local peloid application, sustained H_2_S release	Clinical
**Bone Healing**	↑ Osteogenesis, ↑ mineralization	Osteoblast proliferation, VEGF activation	Preclinical
**Warnings**	Fast-release H_2_S may exacerbate inflammation	Dose- and release- dependent effects on immune cells	Mechanistic studies

**Table 8:** Summary of the main Respiratory/ENT Applications treated with H_2_S, their scientific evidence and the possible mechanism.

Condition	Main Effects	Mechanisms	Evidence
**Allergic & non-allergic rhinitis**	↓ Nasal congestion, ↓ sneezing, ↓ IL-5 and local Ig E	Inhibits Th2 cytokines (IL-5), ↓ IgE; improves epithelial barrier & mucociliary clearance	RCTs + clinical studies
**Chronic pharyngitis/laryngitis**	↓ Inflammation, ↓ dysphonia, ↑ epithelial regeneration	Mucoregulatory, antiseptic, and epithelial-regenerating action	Observational + spa studies
**Chronic bronchitis/mild-to-** **moderate COPD**	↑ FEV_1_, ↓ sputum, ↑ exercise tolerance, ↓ oxidants in exhaled air	↓ ROS; activates Nrf2/HO-1; modulates microbiota & local cytokines	RCTs + systematic review
**Mild persistent/intermittent asthma**	↓ Bronchial hyperreactivity, ↓ inflammation, ↑ lung function	Th2 modulation; ↓ eosinophils; ↓ IL-4/IL-13; activates K^+^ channels & Nrf2	Preclinical + observational spa studies
**Subacute/mild chronic sinusitis**	↑ Mucociliary clearance, ↓ mucus secretion, ↓ inflammation	Improves ciliary transport; ↓ MUC5AC; ↓ microbial biofilm	Observational + physiopathological basis
**Vasomotor/non-allergic rhinitis**	↓ Rhinorrhea, ↑ vascular tone, ↑ ciliary transport	Regulation of neurovegetative tone and nasal secretion	Small clinical studies

**Table 9:** Summary of the main cardiovascular diseases treated with H_2_S, their scientific evidence and the possible mechanism.

Condition	Main Effects	Mechanisms	Evidence
**Vascular tone regulation (NO** **interaction)**	Vasodilation and reduced vascular resistance	K_ATP channel activation → hyperpolarization; ↑ eNOS; nitrosopersulfide formation	Observational + pathophysiological basis
**Ischemia–reperfusion injury**	Mitochondrial protection and antioxidant activity	↑ Nrf2; ↑ HO-1/NQO1; ↓ ROS; persulfidation of CypD	Preclinical + balneary observational
**Myocardial repair**	Increased contractility and angiogenesis	↑ VEGF expression; myocardial angiogenesis	Observational + balneary studies
**Atherosclerosis and inflammation**	Reduced inflammation and LDL oxidation	↓ TNF-α, IL-1β, IL-6, ICAM-1, VCAM-1; ↓ VSMC proliferation	RCT + clinical studies
**Blood pressure control**	Baseline hypertension in CSE^−^/^−^ models	Loss of CSE → ↓ H_2_S → ↑ BP; ↓ endothelial relaxation	Preclinical + balneary observational
**Endothelial epigenetic longevity**	Reduced NF-κB activity and delayed vascular aging	S-sulfhydration of p65 NF-κB → ↓ transcriptional activity	Small clinical studies

**Table 10:** Summary of the main gastrointestinal tract, liver and kidney problems treated with H2S, their scientific evidence and the possible mechanism.

Condition	Main Effects	Mechanisms	Evidence
**Uncomplicated chronic gastritis**	↓ epigastric pain- better gastric tolerance	Local anti-inflammatory effect via H_2_S (↓ NF-κB,↑ Nrf2/SIRT1), mucin stimulation, ↑ mucosal blood flow	Observational clinical studies + solid preclinical
**Peptic ulcer (adjuvant)**	Faster healing, ↓ recurrence	↑ prostaglandins and NO, angiogenesis Activation, ↓ oxidative stress, epithelial repair via H_2_S signaling	Robust preclinical + clinical series
**Functional dyspepsia**	↓ Heartburn, ↓ postprandial fullness	Motility modulation, protection of epithelial tight junctions, local antioxidant action	Small trials + observational
**Functional constipation**	Improved intestinal transit	Stimulation of colonic motility via K_ATP channels and smooth-muscle activation by H_2_S	Preclinical + clinical experience
**General liver function**	Improvement of liver markers (↓ ALT, AST),support for detoxification processes	Activation of endogenous antioxidants (↑ SOD, GPx), reduction of hepatic inflammation and fibrosis via H_2_S	Preclinical studies + observational
**Mild/moderate NAFLD (adjuvant)**	↓ transaminases- slight ultrasound improvement	Systemic antioxidant effect- improved lipid metabolism and insulin sensitivity via H_2_S signaling	Animal studies + human observational
**Metabolic syndrome/Diabetes**	↓ fasting and postprandial glucose- improved insulin sensitivity↓ triglycerides	Activation of redox and metabolic pathways (↑ AMPK), modulation of systemic inflammation	Preclinical + pilot studies
**Hyperuricemia (secondary** **prevention)**	↓ uricemia, ↓ attacks	↑ renal urate excretion via vasodilatory and natriuretic action of H_2_S	Observational; high plausibility
**Uric acid urolithiasis**	↓ recurrence	Urinary alkalinization by suitable mineral composition- renal vasodilation mediated by H_2_S	Observational

**Table 11:** Comparison of how different parameters affect the effectiveness of sulfurous water treatment depending on the use of balneation techniques or the application of peloids.

Characteristics	Sulfurous Peloids	Sulfurous Baths
**Release profile**	Slow and sustained	Rapid and immediate
**Local concentration**	High	More uniform distribution
**Duration of effect**	Prolonged	Limited
**Depth of thermal action**	High	Moderate
**Application temperature**	38–46 °C	34–38 °C
**Additional mineral supply**	Water and peloid	Water
**Scope of action**	Localized	Generalized

**Table 12:** Toxicity of H_2_S, different toxicological parameters from different world toxicology institutions.

Organization	Value Type	Notes	Limit (ppm)
**OSHA (Washington, DC, USA)**	PEL–TWA	Up to 50 ppm allowed if it does not exceed 10 min and never exceeds 20 ppm on average.	20 ppm (ceiling)
**NIOSH (Washington, DC, USA)**	REL–TWA	Recommended limit (8 h/day, 40 h/week).	10 ppm
**NIOSH (Washington, DC, USA)**	REL–STEL	Maximum permitted exposure for 15 min.	15 ppm
**NIOSH (Washington, DC, USA)**	IDLH	Immediately dangerous to life or health.	100 ppm
**ACGIH (Sharonville, OH, USA)**	TLV–TWA	Threshold limit value, 8 h time-weighted average. Revised in 2010.	1 ppm
**ACGIH (Sharonville, OH, USA)**	TLV–STEL	Short-term exposure limit (15 min).	5 ppm
**EU ** **(Brussels, Belgium)**	VLEP–TWA	Occupational exposure limit value (8 h).	5 ppm (7 mg/m^3^)
**EU ** **(Brussels, Belgium)**	VLEP–STEL	Short-term exposure limit value (15 min).	10 ppm (14 mg/m^3^)

Parameters to be determined: PEL (Permissible Exposure Limits); TWA (Time-Weighted Average); REL (Recommended Exposure Limit); STEL (Short-Term Exposure Limit); IDLH (Immediately Dangerous to Life or Health); TLV (Threshold Limit Value): threshold limit value defined by ACGIH; includes TWA (8 h average), STEL (15 min), and Ceiling (ceiling value that must never be exceeded); VLEP (Occupational Exposure Limit Value).

## Data Availability

No new data were created or analyzed in this study. Data sharing is not applicable to this article.

## Abbreviations

The following abbreviations are used in this manuscript:

3-MST	3-mercaptopyruvate sulfurtransferase
ACC	Acetyl-CoA carboxylase
ACGIH	American Conference of Governmental Industrial Hygienists
AE1	Anion Exchanger 1
Akt	Protein kinase B
ALP	Alkaline phosphatase
ALT	Alanine aminotransferase
AMPK	AMP-activated protein kinase
ARE	Antioxidant Response Element
AST	Aspartate aminotransferase
BKCa (KCa1.1)	Large-conductance calcium-activated potassium channel
BMI	Body mass index
*C. acnes*	*Cutibacterium acnes*
CAT	Catalase
CAT (aminotransferase)	Cysteine aminotransferase
CBS	Cystathionine β-synthase
CFTR	Cystic fibrosis transmembrane regulator
CO	Carbon monoxide
COPD	Chronic obstructive pulmonary disease
COX-2	Cyclooxygenase-2
CPT1	Carnitine palmitoyltransferase 1
CPT2	Carnitine palmitoyltransferase 2
CRP	C-reactive protein
CSE	C-reactive protein
DLQI	Dermatology Life Quality Index
DNMTs	DNA methyltransferases
EASI	Eczema Area and Severity Index
eNOS	Endothelial nitric oxide synthase
ERK	Extracellular signal-regulated kinase
EU	European Union
FAO/WHO	Food and Agriculture Organization/World Health Organization
FAS	Fatty acid synthase
FeNO	Fractional exhaled nitric oxide
FEV_1_	Forced expiratory volume in one second
FVC	Forced vital capacity
GABA	Gamma-aminobutyric acid
GCLC	Glutamate–cysteine ligase catalytic subunit
GCLM	Glutamate–cysteine ligase modifier subunit
GGT	Gamma-glutamyl transferase
GPx	Glutathione peroxidase
GSH	Reduced glutathione
GSSG	Oxidized glutathione
GYY4137	Slow-release hydrogen sulfide donor
H_2_S	Hydrogen sulfide
HbA1_C_	Glycated hemoglobin A1c
HDACs	Histone deacetylases
HDL	High-density lipoprotein cholesterol
HO-1	Heme oxygenase-1
HOMAR-IR	Homeostatic Model Assessment of Insulin Resistance
HSP(s)	Heat shock proteins
HS^−^	Hydrosulfide ion
IDLH	Immediately Dangerous to Life or Health
IgE	Immunoglobulin E
IL-1 β	Interleukin-1 beta
IL-4	Interleukin-4
IL-5	Interleukin-5
IL-6	Interleukin-6
IL-8	Interleukin-8
IL-10	Interleukin-10
IL-13	Interleukin-13
IL-17	Interleukin-17
IL-31	Interleukin-31
JAK/STAT	Janus kinase/Signal transducer and activator of transcription
KATP	ATP-sensitive potassium channel
Keap1	Kelch-like ECH-associated protein 1
LDL	Low-density lipoprotein cholesterol
LL-37	Cathelicidin antimicrobial peptide LL-37
MAPK	Mitogen-activated protein kinase
MASLD	Mitogen-activated protein kinase
MDA	Malondialdehyde
mg L^−1^	Milligrams per liter
mg m^−3^	Milligrams per cubic meter
miR-21	MicroRNA-21
MMP(s)	Matrix metalloproteinase(s)
MUC5AC	Mucin 5AC
mTOR	Mechanistic target of rapamycin
NAFLD	Non-alcoholic fatty liver disease
NASH	Non-alcoholic steatohepatitis
NADPH	Nicotinamide adenine dinucleotide phosphate (reduced)
NF- κB	Nuclear factor kappa-B
NIOSH	National Institute for Occupational Safety and Health
NMDA	N-methyl-D-aspartate receptor
NO	Nitric oxide
NO/cGMP	Nitric oxide and cyclic guanosine monophosphate pathway
NQO1	NAD(P)H quinone dehydrogenase 1
Nrf2	Nuclear factor erythroid 2–related factor 2
NSAID(s)	Non-steroidal anti-inflammatory drugs
O_2_	Oxygen
OSHA	Occupational Safety and Health Administration
ORAC	Oxygen Radical Absorbance Capacity
PEF	Peak expiratory flow
PEL	Permissible Exposure Limit
PGC-1α	Peroxisome proliferator-activated receptor gamma coactivator 1-alpha
PI3K	Phosphoinositide 3-kinase
PPAR-α	Peroxisome proliferator-activated receptor-alpha
PPAR-γ	Peroxisome proliferator-activated receptor-gamma
ppm	Parts per million
RCTs	Randomized controlled trials
REL	Recommended Exposure Limit
ROS	Reactive oxygen species
SAA(s)	Sulfur-containing amino acids
SCFAs	Short-chain fatty acids
SCORAD	Scoring Atopic Dermatitis Index
SIRT1	Sirtuin-1
SIRT2	Sirtuin-2
SIRT3	Sirtuin-3
SLC26	Solutes carrier
SOD	Superoxide dismutase
SQR	Sulfide quinone oxidoreductase
SRB	Sulfate-reducing bacteria
SREBP-1c	Sterol regulatory element-binding protein-1c
S^2−^	Sulfide ion
STEL	Short-Term Exposure Limit
TAC	Total antioxidant capacity
TGF-β	Transforming growth factor beta
TEWL	Transepidermal water loss
Th1	T helper 1
Th2	T helper 2
Th17	T helper 17
TLR4	Toll-like receptor 4
TLV	Threshold Limit Value
TRP	Transient receptor potential
TRPA1	Transient receptor potential ankyrin 1
TRPV1	Transient receptor potential vanilloid 1
TSLP	Thymic stromal lymphopoietin
TG	Triglycerides
TWA	Time-Weighted Average
VEGF	Vascular endothelial growth factor
VLEP	Occupational Exposure Limit Value (EU)
Wnt/β-catenin	Wnt signaling/β-catenin pathway
°C	Degrees Celsius
